# Temporal changes in pathology and viral RNA distribution in guinea pigs following separate infection with two New World Arenaviruses

**DOI:** 10.1371/journal.pntd.0011620

**Published:** 2023-09-08

**Authors:** Curtis Cline, Xiankun Zeng, Todd M. Bell, Carl Shaia, Paul Facemire, Janice Williams, Neil Davis, April Babka, Edwin Picado, Colin Fitzpatrick, Joseph W. Golden

**Affiliations:** 1 Pathology Division, United States Army Medical Research Institute of Infectious Diseases, Fort Detrick, Maryland, United States of America; 2 Foundational Sciences Directorate, United States Army Medical Research Institute of Infectious Diseases, Fort Detrick, Maryland, United States of America; 3 Rocky Mountain Veterinary Branch, Division of Intramural Research, National Institute of Allergy and Infectious Diseases, National Institutes of Health, Hamilton, Montana, United States of America; 4 Virology Division, United States Army Medical Research Institute of Infectious Diseases, Fort Detrick, Maryland, United States of America; Karolinska Institutet, SWEDEN

## Abstract

Numerous arenaviruses have been identified throughout the Americas and a subset of these viruses cause viral hemorrhagic fever in humans. This study compared the pathology and viral RNA distribution in Hartley guinea pigs challenged with two human-disease causing New World arenaviruses, Junin virus (JUNV) or Guanarito virus (GTOV). Histopathologic analysis and RNA in situ hybridization revealed similar pathology and viral RNA distribution for both groups of animals challenged with either JUNV or GTOV on days 3, 7, 10 and 12 post exposure (PE). Gross lesions were first observed on day 7 and primarily involved the lungs and liver. The most severe histologic lesions occurred in the lymph nodes, spleen, and thymus and included lymphoid depletion and necrosis which increased in severity over time. Extensive necrosis was also observed in the bone marrow on day 12. Minimal to mild inflammation with and without necrosis was observed in the choroid plexus of the brain, choroid of the eye, intestinal tract, lung and adrenal gland. Significant liver lesions were rare, consisting predominantly of hepatocyte vacuolation. Viral RNA labeling was identified in nearly all organs examined, was often extensive in certain organs and generally increased over time starting on day 7. Our data demonstrate the guinea pig may serve as a useful model to study New World arenavirus infection in humans and for the evaluation and development of medical countermeasures.

## Author summary

Arenaviruses are a type of RNA virus that primarily exist within rodent reservoirs, which are often virus specific. The family of arenaviruses are divided into two large groups, new world and old world, based upon the geographic region where they were initially isolated. A subset of arenaviruses are capable of being transmitted to humans, where they may cause viral hemorrhagic fever (VHF), a condition characterized by multiorgan system involvement, immune and vascular system dysregulation and that ultimately results in bleeding/hemorrhage and can lead to death. Licensed vaccines and therapies do not exist for many VHF causing arenaviruses. Two such viruses include Junin virus and Guanarito virus. Guinea pigs have been previously used as a model to study disease pathogenesis following new world arenavirus infection. Herein we describe in detail and compare the organ system specific pathology and viral RNA distribution following separate infection in Guinea pigs, with Junin virus and Guanarito virus. This study provides valuable insight into the time course of infection, viral target tissues and similarities between two different new world arenaviruses, that will ultimately aid in development of therapies that may be effective across the spectrum of new world arenaviruses.

## Introduction

Arenaviruses are enveloped ambisense single-stranded RNA viruses that are maintained in nature by persistent infections in rodent species. This family is divided into two general complexes, old world (OW) and new world (NW), based on initial geographical region of virus isolation. Of the more than 20 known species of arenaviruses only a select few cause disease in humans [1]. The most prominent human pathogen is Lassa virus (LASV), an OW arenavirus which causes Lassa Fever (LF) resulting in 100-300K human infections per year. New World arenavirus clades include A, B A/B and C. This manuscript focuses on JUNV and GTOV, both members of clade B, and both known to cause viral hemorrhagic fever in humans, with JUNV resulting in Argentine hemorrhagic fever (AHF) and GTOV resulting in Venezuelan hemorrhagic fever (VHF) [2]. These two viruses are included in a group of NW arenaviruses occurring in endemic regions throughout South America and extending as far north as the southwestern United States, where potential illness has been reported from infection with a New World arenavirus [1,3–5]. In addition to GTOV and JUNV, lineage/clade B includes other pathogenic viruses such as Machupo (MACV) and Sabia (SABV) viruses which cause Bolivian hemorrhagic fever (BHF) and Brazilian hemorrhagic fever (BzHF) respectively, as well as Chapare virus infection, which also occurs in Bolivia [5,6].

NW Arenavirus cases generally occur in low numbers and case fatality rates vary by agent, but are reportedly as high as 30% in AHF without treatment [5,7]. Additionally, because of their route of transmission and potential to cause severe and fatal disease with significant public health impact, arenaviruses have been classified as category A Bioterrorism agents by the U.S. Centers for Disease Control and Prevention [8]. The primary route of transmission is by inhalation or ingestion of contaminated urine or feces shed by the rodent reservoir, the Vesper mouse (JUNV) and short-tailed cane mouse (GTOV). [3,5]. Person to person transmission has been reported with NW arenaviruses but is considered uncommon [1,9].

The human clinical and pathological findings caused by NW arenavirus infection are mostly reported based on AHF, the existence of which has been known for a greater amount of time and has been studied more extensively. Nonetheless, many of the clinical features are generally true for what has been reported for VHF [10]. Infection may begin in the lung due to inhalation of infected particles and deposition in terminal bronchioles, followed by infection of macrophage/monocyte lineage cells, early replication in lung tissues, infection of the lymphoid system and systemic spread [10,11]. The incubation period lasts approximately 1–2 weeks and starts with many non-specific symptoms (i.e. fever, malaise) but may also include lymphadenopathy, mucosal petechiae, thrombocytopenia, and leukopenia which is followed by and may progress to respiratory distress, CNS symptoms, and gastrointestinal and cardiac involvement [2,10–12]. Disseminated intravascular coagulation is an uncommon finding but has been reported along with autopsy findings of fibrin thrombi [13]. There are often alterations in coagulation factors reported during the course of disease, but the severity is not correlated with the severity of disease [10,11,14]. Infection of endothelial cells also occurs [10]. The later symptoms associated with hemorrhagic fever may include shock, hemorrhage in various locations such as the gastrointestinal tract as well as neurological involvement/coma, which may predominate [2,10,11,13].

Findings reported at autopsy in cases of VHF include pulmonary and renal edema, splenomegaly and hemorrhages in multiple organs including the lung, liver, epicardium, gastrointestinal tract, urinary bladder and uterus [15]. Lesions in fatal cases of human AHF may include hemorrhage in multiple organs, renal tubular and papillary necrosis and focal or multifocal necrosis of individual hepatocytes, myocarditis and secondary bacterial infections [11,13]. In spite of neurologic symptoms associated with the clinical syndrome, nervous system pathology is not commonly described and the origin of the neurological component of the disease remains unclear [11]. Central nervous system findings that have been uncommonly reported include hemorrhage in Virchow Robin space, perivascular infiltrates and microglial proliferation [13]. Importantly, fatal cases of AHF are also associated with hematolymphoid tissue lesions such as white pulp necrosis in the spleen with lymphocyte depletion, scattered necrosis in the red pulp, and lymphocyte depletion in the cortex/paracortex of lymph nodes with pyknotic lymphocytes, as well as depletion of multiple cell lines in the bone marrow [11,16]. These clinical and pathological findings create a complex picture of hemorrhagic fever caused by NW arenaviruses for which animal models will facilitate a better understanding, ultimately leading to a more thorough understanding of the infection dynamics that result in illness and death.

Correlating the pathology findings with viremia data and cytokine analysis is also crucial to understanding the immunosuppressive aspects of NW arenavirus infection. Comparison of these findings across all pathogenic NW arenaviruses will advance our understanding of the potential for cross protection of vaccines and therapeutics. Herein we compare the pathology and viral RNA distribution pattern caused by JUNV infection or GTOV infection in a Harley (outbred) Guinea pig model. We show that many of the pathologic features associated with NW arenavirus infection, as well as cytokine levels, are very similar in JUNV and GTOV infection, which may support development of cross protecting medical countermeasures for this group of viruses. Previous studies have shown that mouse and non-human primate models are useful to study specific aspects of the immune response to viral infection and vaccines/therapeutics respectively [5,7,17–19]. However, guinea pigs have proven to serve as an optimal model able to mimic important pathologic aspects of human disease while simultaneously facilitating the study of temporal disease development and disease pathogenesis [5,7,20–22]. Our findings show that targeting of hematolymphoid organs is an important aspect of infection and that the vascular endothelium and brain appear to be early targets of infection but do not result in significant pathology prior to day 12. We also reveal important findings about the timing of infection related events and demonstrate that the patterns of lesion development and infection are very similar between JUNV and GTOV thereby providing clarity regarding the disease progression of two important NW arenaviruses.

## Materials and methods

### Ethics statement

Research was conducted under an Institutional Animal Care and Use Committee (IACUC) approved protocol in compliance with the Animal Welfare Act, Public Health Service (PHS) Policy on Humane Care and Use of Laboratory Animals, and other Federal statutes and regulations relating to animals and experiments involving animals. The facility where this research was conducted is accredited by the Association for Assessment and Accreditation of Laboratory Animal Care (AAALAC), International and adheres to principles stated in the Guide for the Care and Use of Laboratory Animals, National Research Council, 2011. Animals meeting pre-established criteria were humanely euthanized in accordance with American Veterinary Medical Association guidelines.

### NW arenavirus guinea pig challenge

Female Hartley guinea pigs (300-400g; Charles River Laboratories) were infected with 2,000 PFU of JUNV or GTOV by the intraperitoneal (IP) route (16/group). Guinea pigs were monitored for clinical signs of disease (ruffled fur, hunched posture) and weighed daily. At four time points (+3, +7, +10 and +12 days post infection) based on the time course of disease, four animals per group were euthanized (Euthasol) subsequent to anesthesia. There were no unscheduled necropsies. Three animals were designated as uninfected controls and were euthanized on day 12 subsequent to anesthesia. Blood samples (serum) were collected prior to euthanasia and assayed for viral and cytokine levels. Necropsies were conducted and tissues collected for histopathology and in-situ hybridization for viral RNA. Tissues examined include the brain, lungs, heart, thymus, trachea, esophagus, thyroid gland, adrenal gland, liver, spleen, mesenteric and mandibular lymph nodes, salivary gland, bone marrow, pancreas, stomach, small intestine, large intestine, kidney, urinary bladder, ovaries, uterus, eyes, and skeletal muscle.

### Viruses

JUNV strain Romero [23] and GTOV strain INH95551 [20] were propagated on Vero cell monolayers (ATCC CRL-1587) in Eagle minimal essential medium, containing 10% heat-inactivated fetal bovine serum (FBS), 1% antibiotics (100 U/ml penicillin, 100 mg/ml of streptomycin), and 10 mM HEPES (cEMEM) as previously described [24]. All virus work was conducted in a BSL-4 containment laboratory at United States Army Medical Research Institute of Infectious Diseases (USAMRIID) that was fully compliant with applicable federal statutes.

### Serum viremia

Viremia was quantified by plaque assay essentially as previously described [25] with some variation. Sera samples were serially diluted 10-fold in cMEM. Subsequently, 100 μl of sample was adsorbed to confluent Vero cell monolayers in 6-well plates for 1 h in a 37°C 5% CO_2_ incubator and rocked ~15 m. Following adsorption, a 2 ml solid overlay (Earle’s basal minimal essential medium (EBME), 0.5% agarose, 5% heat inactivated FBS, antibiotics (100 U/ml penicillin, 100 μg/ml of streptomycin, and 50 μg/ml of gentamicin) was added to each well. Plates were incubated for six days in a 37°C 5% CO_2_ incubator, 80–85% humidity then stained with 2 ml of solid overlay mixture that also included 5% neutral red (Gibco). Cells were incubated an additional 24 h in a 37°C 5% CO_2_ incubator before plaque counting. Data were plotted using Prism software.

### IFN-α levels

Levels (pg/ml) of serum IFN-α (100 μL per sample) were determined by commercial ELISA (ABClonal; Cat#RK00743) according to the manufacturer’s directions. Uninfected guinea pigs (n = 3) were used as a baseline control.

### Gross necropsy and histology

All necropsies were performed by a histopathology technician and/or a board-certified veterinary pathologist (day 10 and 12 animals) within 3 hours of euthanasia or estimated time of death in the USAMRIID BSL-4 laboratory. The tissues were fixed by immersion into labeled containers of 10% neutral buffered formalin and held in biocontainment for a minimum of 21 days. The tissues were routinely trimmed, processed, embedded in paraffin, cut by microtomy, stained, cover-slipped and screened. All slides were examined by the same board-certified veterinary pathologist. Histopathology and In-situ hybridization severity scores were determined based on the following criteria: Minimal if 10% or less of the cells in the section are affected, then score is 1; Mild if between 11% and 25% of the cells in the section are affected, then score is 2; Moderate if between 26% and 50% of the cells in the section are affected, then score is 3; Marked if between 51% and 79 of the cells in the section are affected, then the score is 4; Severe if between 80% or more the cells in the section are affected, then the score is 5.

### In situ hybridization

To detect Junin virus (JUNV) and Guanarito virus (GTOV) genomic RNA, RNA *in situ* hybridization (ISH) was performed using the RNAscope 2.5 HD RED kit (Advanced Cell Diagnostics, Newark, CA, USA) according to the manufacturer’s instructions. Briefly, an ISH probe targeting the reverse complement sequence of 56–1681 of JUNV segment S (genbank accession number JN801476.1) or the reverse-complement sequence of 7–1664 of GTOV segment S (genbank accession number AY129247.1) was designed and synthesized by Advanced Cell Diagnostics. Tissue sections were deparaffinized with xylene, underwent a series of ethanol washes and peroxidase blocking, and were then heated in kit-provided antigen retrieval buffer and digested by kit-provided proteinase. Sections were exposed to ISH target probe pairs and incubated at 40°C in a hybridization oven for 2 h. After rinsing, ISH signal was amplified using kit-provided Pre-amplifier and Amplifier conjugated to alkaline phosphatase and incubated with a Fast Red substrate solution for 10 min at room temperature. Sections were then stained with hematoxylin, air-dried, and mounted.

To detect guinea pig cytomegalovirus (GPCMV), ISH was performed as described above using a previously published ISH probe [26] (Cat# 826061 Advanced Cell Diagnostics) targeting the mRNA transcript *gp3* of GPCMV [26].

Statistical analysis. Levels of IFN-α were compared between the different groups using two-way ANOVA.

## Results

**Fig 1:**
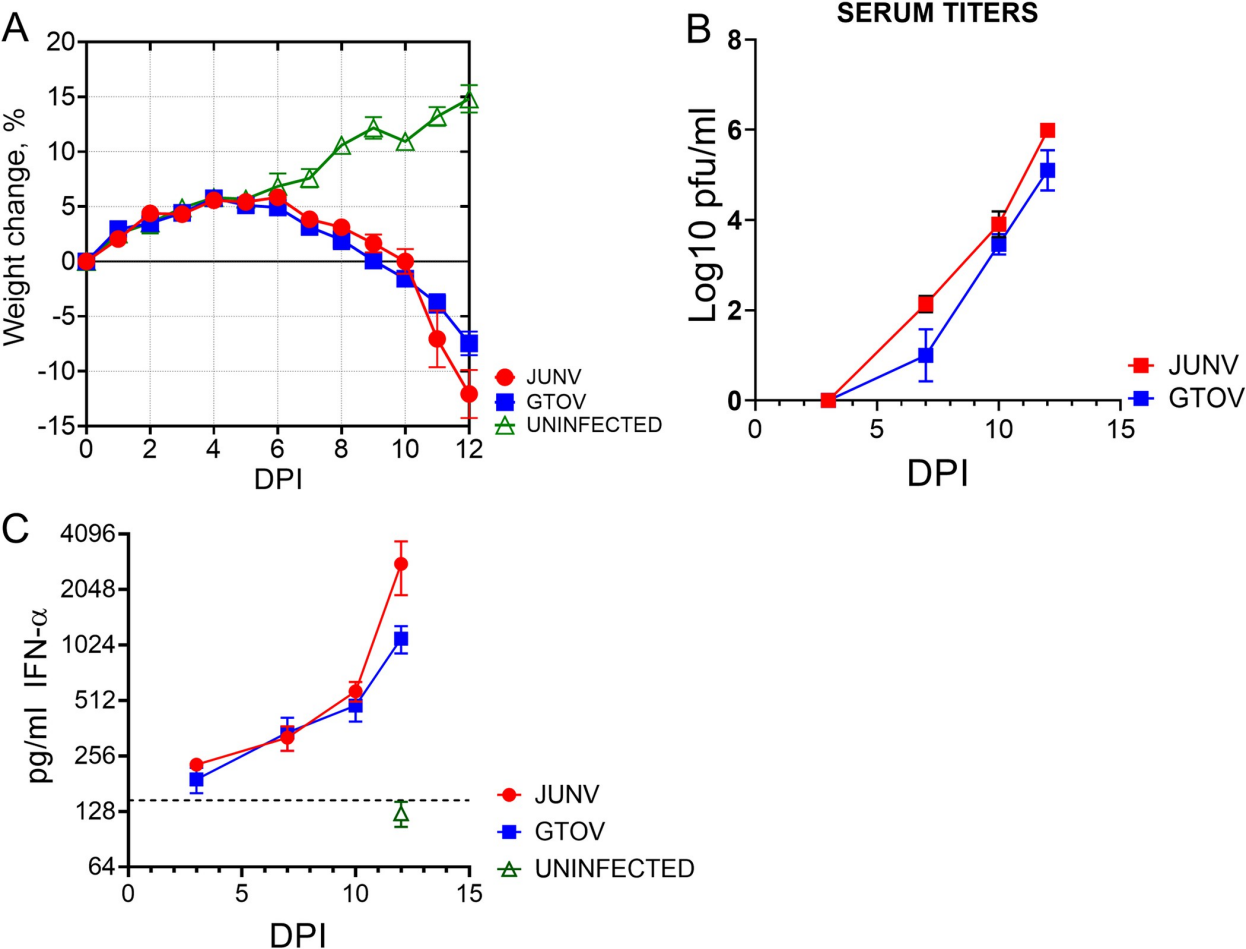
Weight change, viremia and serum IFN-α in JUNV and GTOV infected guinea pigs. Guinea pigs (n = 16 per virus) were infected with the indicated viruses at 2,000 PFU by the IP route. Three uninfected animals were included as controls. A. Weight loss was monitored over the course of the study and individual weights were calculated based on Day 0 starting weight. B. Viremia (n = 4 guinea pigs per virus per time point) was determined by plaque assay. Log10 titer (pfu/ml) were calculated and graphed with standard deviation. C. Levels of serum IFN-α were determined by guinea pig-specific ELISA. Uninfected guinea pigs (N = 3) were used as a baseline control. Dashed line indicates mean titer of uninfected animals plus three standard deviations.

Two groups of guinea pigs (n = 16/group) were challenged with 2,000 pfu of JUNV or GTOV by the IP route. Three uninfected Hartley guinea pigs were used as controls for this study. Animals were monitored daily for weight loss (Fig 1A). Animals in both groups gain weight between days 0 and 4, and then begin to lose weight on day 6 through day 12, versus uninfected control animals which continued to gain weight throughout the study (Fig 1A). Weight loss was similar between JUNV and GTOV infected animals. By day 12 JUNV infected guinea pigs lost 12% and GTOV lost 7.5% of their body weight compared to day 0 (Fig 1A). On days 3, 7, 10 and 12 four infected guinea pigs per group were euthanized for sample collection. The three uninfected animals were euthanized on day 12. Serum viremia was evaluated by plaque assay. No virus was detected in either group on day 3; however, titers were detectable on day 7 and increased through day 12, culminating in ~6 log pfu/ml for JUNV and 5 log pfu/ml for GTOV. We also evaluated the level of serum IFN-α by ELISA. IFN-α levels were above baseline starting on day 3 and continued to increase in both groups over the course of infection. By day 12, JUNV had the highest levels of IFN- α with a median level of 2,818 pg/ml versus GTOV at 1,107 pg/ml (Fig 1C). The difference between JUNV and GTOV IFN-α levels were statistically significant on day 12 (2-way ANOVA; p = 0.0054).

### Gross pathology

Gross lesions were generally mild and limited to observations in the lymph nodes, lung and liver. Gross lesions were absent in day 3 animals from both groups. Gross lesions that may be attributable to arenavirus infection in the JUNV and GTOV infected day 7 animals included red, diffusely mottled discoloration in the lungs affecting all lobes; enlarged mandibular and mesenteric lymph nodes approximately two to three times normal size; and multifocal, pinpoint areas of tan discoloration in the liver. Gross lesions in both the JUNV and GTOV infected day 10 animals, included multifocal, mild, patchy red discoloration in all lung lobes. The JUNV infected day 10 animals also had multifocal, minimal, small pin-point areas of tan discoloration in the liver. Gross lesions in both the JUNV and GTOV infected day 12 animals and the control animals included multifocal, mild, patchy red discoloration in all lobes. The JUNV and GTOV infected day 12 animals also had multifocal, small (less than 0.5cm) areas of tan discoloration in the liver. A single GTOV infected day 12 animal also had larger (0.5–1 cm), patchy to linear, areas of darker brown discoloration in the liver. No significant gross lesions were identified in the brain, eyes, thymus, heart, trachea, esophagus, salivary glands, thyroid gland, spleen, small and large intestines, adrenal glands, pancreas, gastrointestinal tract, skeletal muscle, kidney, urinary bladder or reproductive organs.

### Histology & ISH

**Fig 2:**
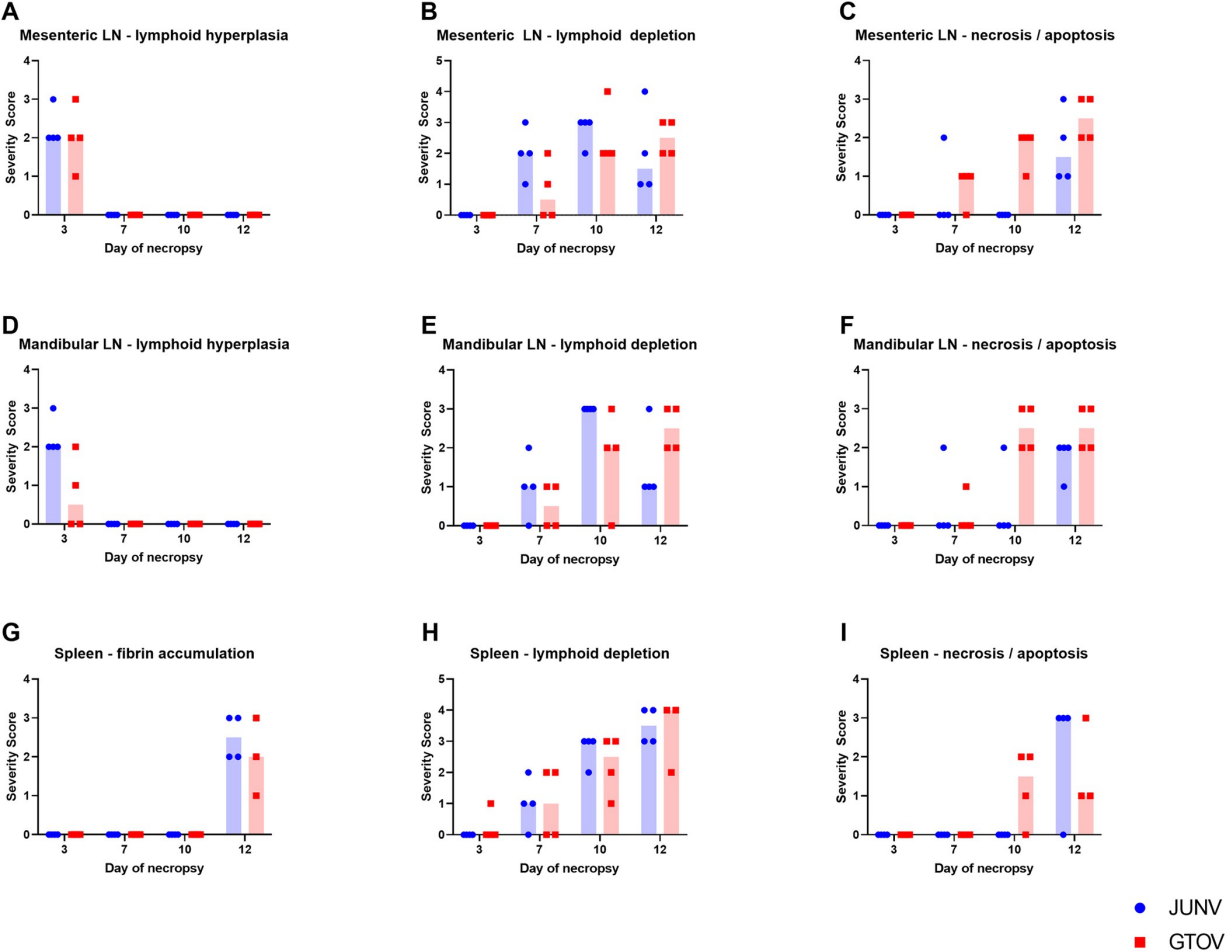
Temporal severity of histology lesions in lymph nodes and spleen for both JUNV and GTOV groups. In graphs A–I select histology lesions are graphed for the lymph nodes and spleen to display changes in lesion severity over time between JUNV and GTOV. A single dot or square represents a single animal and the blue or red shaded region represents the median.

The organs with the highest severity scores and the greatest constellation of lesions were the hematolymphoid organs. Organs examined include the mesenteric and mandibular lymph nodes (LNs), spleen, thymus and bone marrow. Significant histopathology and ISH findings are summarized by number of animals affected at each time point, for each group, in Table 1 (table of findings is not comprehensive). Findings in the lymph nodes included lymphoid hyperplasia in both the follicles and paracortical regions. This finding was seen exclusively in a subset of day 3 animals in both lymph nodes and in both JUNV and GTOV infected groups (Fig 2A and 2D). There was mild variation in the number of animals affected for each lymph node, severity scores and/or whether the lymphoid hyperplasia was predominantly follicular or paracortical, but otherwise the lesions were similar between groups.

**Table 1:** Summary of major histopathology and ISH findings by number of animals affected, for both viruses, at each time point.

Organ	Lesion	JUNV				GTOV			
		Day 3	Day7	Day10	Day12	Day 3	Day7	Day10	Day12
Mesenteric Lymph node	lymphoid hyperplasia^a^	4	0	0	0	4	0	0	0
	lymphoid depletion	0	4	4	4	0	2	4	4
	apoptosis/necrosis	0	1	0	4	0	3	4	4
	HEV / vessel hypertrophy	0	3	4	4	0	3	3	4
	ISH positive	1	4	4	4	0	4	4	4
Mandibular lymph node	lymphoid hyperplasia	4	0	0	0	2	0	0	0
	lymphoid depletion	0	3	4	4	0	2	3	4
	apoptosis/necrosis	0	1	1	4	0	1	4	4
	HEV / vessel hypertrophy	0	3	4	4	0	4	3	4
	ISH findings	0	4	4	4	0	4	4	4
Spleen	lymphoid depletion	0	3	4	4	1	2	4	3^c^
	Red pulp infiltrate	0	3	4	3	1	4	4	3^c^
	apoptosis/necrosis^b^	0	0	0	3	0	0	3	3^c^
	fibrin accumulation	0	0	0	4	0	0	0	3^c^
	ISH findings	0	4	4	4	0	4	4	3^c^
Thymus	lymphocyte apoptosis	0	1	1^c^	4	0	0	3	4
	ISH findings	0	4	3^c^	4	0	4	4	4
Bone marrow	necrosis	0	0	2	4	0	0	2	4
	hemorrhage	0	0	0	4	0	0	0	2
	fibrin accumulation	0	0	0	4	0	0	0	2
	ISH findings	0	4	4	4	0	4	4	4
Brain	choroid plexus (CP) necrosis	0	0	0	1	0	0	0	0
	CP inflammation	0	0	0	3	0	0	0	1
	ISH findings	0	0	4	4	0	0	4	4
Eye	choroid inflammation	0	0	0	4	0	0	0	1
	choroid necrosis	0	0	0	2	0	0	0	1
	conjunctival lymphoid necrosis/apoptosis	0	0	0	2	0	0	0	0
	ISH findings	0	1	4	4	0	1	3^c^	4
Liver	vacuolation	4	1	1	4	3	1	2	4
	ISH findings	0	4	4	4	0	4	3	4
Small intestine^d^	GALT hyperplasia	2	0	2	1	2	3	0	1
	lamina propria necrosis/apoptosis	0	0	0	4	0	0	4	4
	GALT necrosis	0	0	0	1	0	0	1	1
	ISH findings	0	3	4	4	0	4	4	4
Adrenal gland	cortical necrosis	0	0	0	1	0	0	1	1
	cortical hemorrhage	0	0	0	4	0	0	0	0
	cortical inflammation	0	0	0	1	0	0	0	2
	ISH findings	0	1	4	4	0	2	4	4

Total number for all animals for each time point, for each group, was four unless otherwise indicated

^a^ Lymphoid hyperplasia includes follicular, paracortical or both

^b^ Refers to necrosis or apoptosis in the red and/or white pulp

^c^ This tissue was omitted from harvest in one animal so total number = 3

^d^ Includes lesions present in any section of the duodenum, jejunum and/or ileum

HEV = high endothelial venule

CP = choroid plexus

GALT = gut associated lymphoid tissue

### Hematolymphoid system

**Fig 3:**
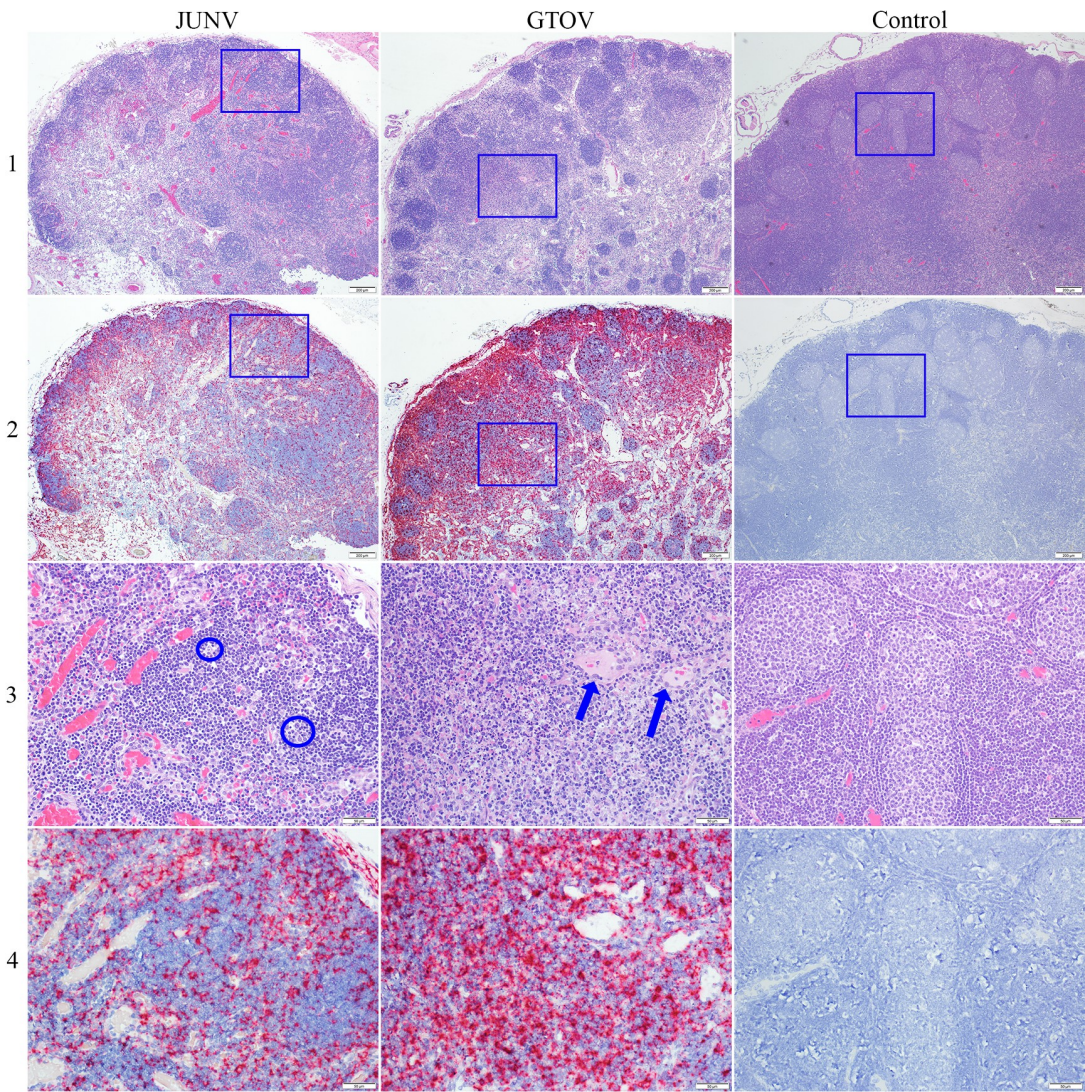
Comparison of the mandibular lymph node (LN) of day 12 JUNV and GTOV infected animals. Blue boxes in rows 1 and 2 represent enlarged areas in rows 3 and 4. Row 1 represents H&E at 4X magnification; note the decreased density of lymphocytes in the cortex (and medullary cords), including follicles and parafollicular areas. The control LN is mildly hyperplastic with follicles containing prominent germinal centers. Row 2 represents ISH at 4X magnification and shows marked labeling of viral RNA (in same area shown in row 1) in the depleted lymph nodes including cortex, medullary cords, and prominent outlining of vessels in the GTOV group. Row 3 represents H&E at 20X magnification; there is depletion of lymphocytes with apoptosis/necrosis, which is more prominent in the GTOV image, increased tingible body macrophages (blue ovals) and vessels with hypertrophied endothelium (blue arrows). Row 4 represents ISH at 20X magnification; there is marked viral RNA labeling in same area shown in row 3, with labeling throughout the cortex including in areas of apoptosis/necrosis and in vessels.

Lymphoid depletion was consistently observed in both the mandibular and mesenteric lymph nodes on days 7, 10 and 12 in both JUNV and GTOV infected animals (Figs 2B, 2E and 3). Depletion increased in severity on days 10 and 12 compared to day 7, and this pattern was more consistent in the GTOV infected animals. The lesion is characterized by decreased density of lymphocytes in follicles and paracortical areas and was often accompanied by an increase in tingible body macrophages. Lymphoid depletion occasionally resulted in the absence of germinal centers and/or follicles highlighting a prominent reticular meshwork. Lymphoid necrosis/apoptosis was also present in both lymph nodes on days 7, 10 and 12, in both the JUNV and GTOV infected animals (Fig 2C and 2F). There was variation in this lesion frequency and severity between groups across all days but the only consistent pattern which emerges is the lesion frequency and severity appear greater on day 10 in the GTOV infected animals compared to the day 10 JUNV infected animals (Figs 2C, 2F and 3). Lymphoid necrosis/apoptosis was characterized by either small multifocal to regionally extensive areas of lymphocyte apoptosis with nuclear pyknosis, karyorrhexis and apoptotic bodies and an absence of accompanying inflammation. The lesion was primarily in the cortical and paracortical region of lymph nodes and occasionally in the medullary cords. Areas of lymphocyte apoptosis/necrosis are often populated with numerous tingible body macrophages phagocytosing apoptotic debris and often coincides with the above described lymphocyte depletion and thus, the underlying stroma is occasionally prominent with scattered apoptotic debris.

Infiltrates of histiocytes were present in the sinuses in both LNs, most often medullary but occasionally cortical, in both the JUNV and GTOV infected groups. The lesion was noted in a subset of animals at the majority of time points for both groups and there was mild variation when comparing groups across the different lymph nodes and time points. The presence/absence and severity were most similar on days 7 and 10, and most dissimilar on day 12, where the lesion was more common in GTOV infected animals (i.e. present in 0–25% of day 12 JUNV infected animals versus 75–100% of day 12 GTOV infected animals). Of note, the lesion was also present in two of three controls in the mesenteric lymph node suggesting at least to some degree it may represent a background finding. Perivascular hemorrhage was also observed in day 7 and 10 animals, predominantly in the mandibular lymph node. While present in both JUNV and GTOV infected animals, it was more common in GTOV infected animals with 62.5% of day 7/10 animals affected and only 25% of JUNV animal affected. The lesion was characterized by extravasation of erythrocytes, often in multiple layers in the cortex, surrounding small or intermediate caliber vessels, with hypertrophic or reactive endothelium.

Vascular changes in the cortex, paracortex and medulla were observed in multiple lymph nodes and most resembles hypertrophy of high endothelial venules (HEV) [27] but also included hypertrophy/reactivity of vascular endothelium in other vessels, such as those in the medullary cords. This finding was present in both JUNV and GTOV infected animals with similar severity and distribution across days 7, 10 and 12, but not day 3 and was characterized by hypertrophy of vascular endothelium, either of the normally larger endothelium of HEVs or the endothelium of other vessels (Fig 3, row 3)). Additional accompanying features include increased migration of lymphocytes across vessel walls, increased aggregation of lymphocytes around vessels and dilation of vessels. These changes occur in response to immune stimulation (i.e. cytokines from the LN stroma) which increases migration of lymphocytes and may be associated with increased cellularity in the lymph node [27].

**Fig 4:**
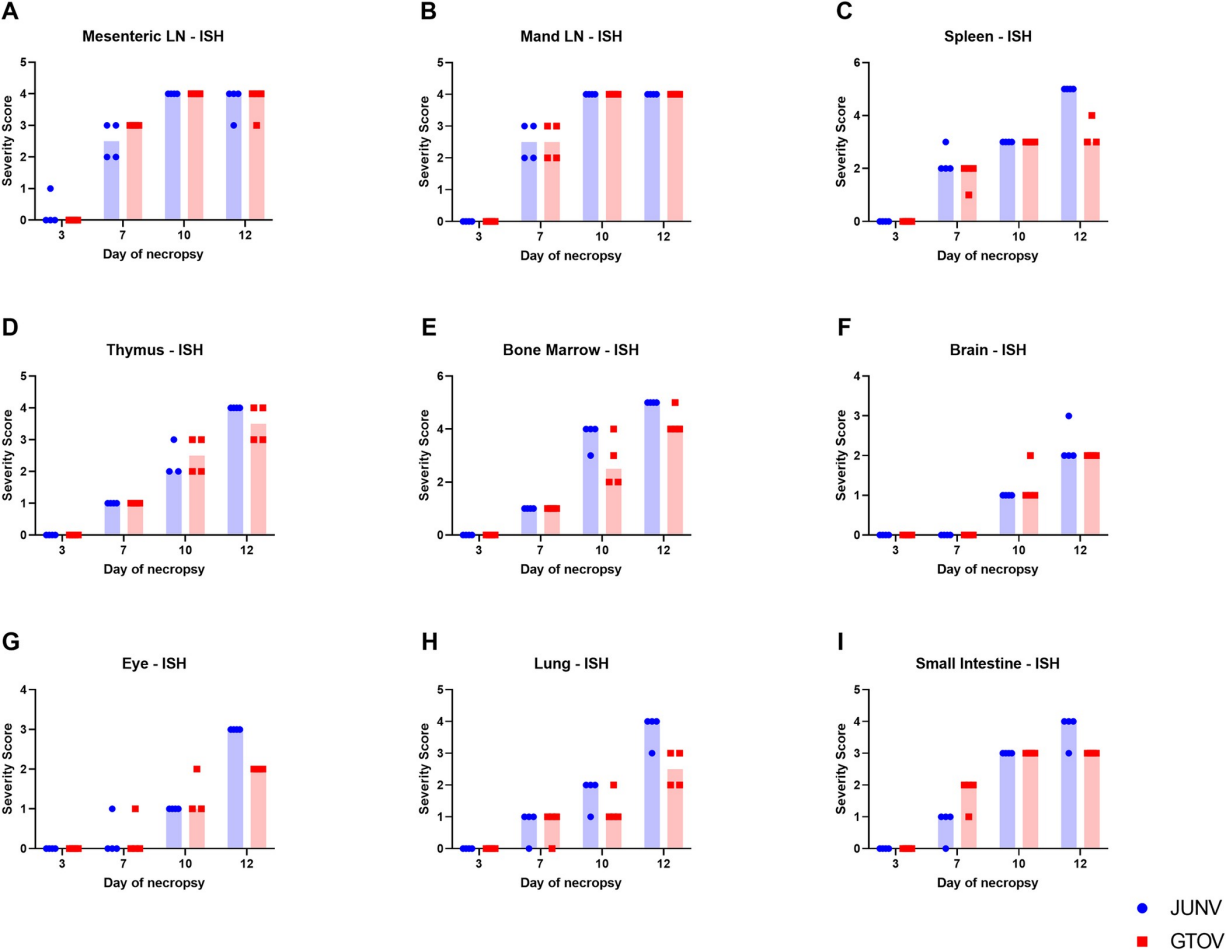
Temporal severity of ISH/viral RNA labeling in select organs for both JUNV and GTOV groups. In graphs A–I severity of viral RNA labeling is graphed for select organs to display changes in labeling severity over time between JUNV and GTOV. A single dot or square represents a single animal and the blue or red shaded region represents the median.

*In situ* hybridization (ISH) results in the lymph nodes for JUNV and GTOV infected animals were similar in severity, spatial and temporal distribution across days (Fig 4A and 4B). ISH results are summarized by number of animals affected for major organs, by group and time point, in Table 1. The primary differences, albeit minor, include more prominent/frequent labeling in medullary vessels and sinuses in GTOV infected animals at days 10 and 12 than in JUNV animals in some cases, but this is a subtle distinction (Fig 3, row 2). One additional difference is that minimal labeling was noted in a single day 3 animal in the JUNV-infected group but none of the day 3 GTOV infected animals. Both lymph nodes also showed a similar pattern of labeling. There was a clear increase in labeling from days 7 to 10 but labeling was relatively similar on days 10 and 12. Regions with labeling include the cortex (often surrounding follicles but with less labeling in follicles), paracortex and medullary cords with the most extensive labeling often being in the cortex. Labeling was frequently strong and prominent with the strong signal often preventing the ability to determine with precision the cell type infected in many areas. Nonetheless, labeling was specifically identified in macrophages in sinuses, cortical vessels and HEVs, and small and large medullary vessels, including the tunica adventitia of some arteries. Labeling was also present in sinus lining cells (i.e. lymphatic endothelium and/or macrophages). In addition to labeling in the lymph node parenchyma, there was frequently strong labeling in the lymph node capsule and vessels and connective tissue of the surrounding peri-nodal adipose tissue, particularly on days 10 and 12. Additional techniques (i.e. IFA, duplex or multiplex) would be necessary to distinguish labeling in lymphocytes, antigen presenting cells (APC), stromal cells such as fibroblastic reticular cells (FRCs) and macrophages in the cortex, paracortex and medullary cords.

**Fig 5:**
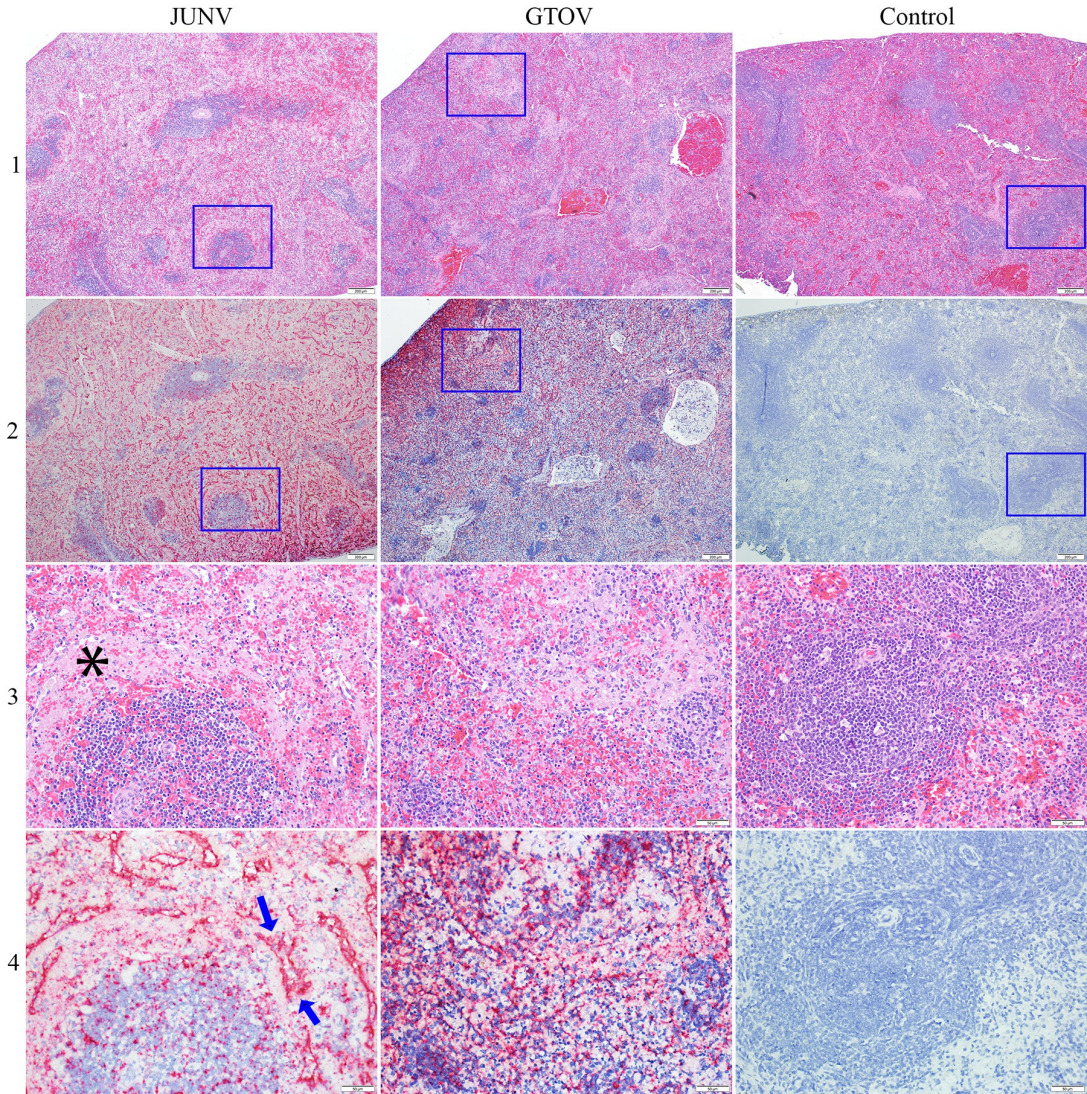
Comparison of the spleen of day 12 JUNV and GTOV infected animals. Blue boxes in rows 1 and 2 represent enlarged areas in rows 3 and 4. Row 1 represents H&E at 4X magnification; note decreased density of lymphocytes in white pulp, including follicles, PALS and marginal zone (most obvious in GTOV animal). Control spleen shows normal red pulp and white pulp architecture and lymphocyte density. Row 2 represents ISH at 4X magnification; there is marked labeling of viral RNA (in same area as shown in row 1) in both the white and red pulp, with prominently outlining red pulp vascular spaces. Row 3 represents H&E at 20X magnification; there is decreased density of lymphocytes in white pulp (nearly absent in GTOV animal), abundant deposition of fibrin in the marginal zone of the white pulp (black asterisk) and in the red pulp, scattered apoptotic/necrotic debris and hemorrhage in the white pulp. Row 4 represents ISH of spleen at 20X magnification; marked viral RNA labeling is present (same area shown in row 3), with labeling in white and red pulp and prominent outlining of red pulp vascular spaces (between blue arrows).

Many of the lesions noted in the lymph nodes such as lymphoid depletion and apoptosis/necrosis were also present in the spleen. The severity scoring and temporal distribution of lesions in the spleen were similar between the JUNV and GTOV infected animals. There was minimal lymphoid depletion in one day 3 animal in the GTOV group but and none of the day 3 JUNV group. Lymphoid depletion in the white pulp was noted predominantly at days 7, 10 and 12, with an increase in severity scoring at later time points (Figs 2H and 5). The lesion was characterized by a decrease in lymphocyte density in the white pulp, follicles, including the marginal zone, and Periarterial lymphatic sheath (PALS) in most cases. Occasionally lymphocyte depletion predominated in the PALS. Similar to the lymph nodes, apoptosis/necrosis was also observed in the splenic white and occasionally red pulp. Lymphoid depletion and apoptosis/necrosis were inconsistent in severity and frequency and differed between groups (Fig 2I). In the JUNV animals, lymphoid apoptosis/necrosis was present in three day 12 animals in both the red and white pulp (Figs 2I and 5). Whereas in the GTOV-infected animals the lesion was present in day 10 as well as day 12 animals with greater variation in severity, and was predominantly present in the white pulp in the majority of animals (67% of those affected). Splenic lymphoid apoptosis/necrosis was generally more common in the GTOV infected animals, but less severe than in the JUNV infected animals and was characterized by karyorrhectic and pyknotic nuclear lymphocyte debris scattered throughout the red and/or white pulp (PALS, follicles, and/or marginal zone), often within macrophages. Occasionally the karyorrhectic debris was dense, but in the an absence of inflammation, most consistent with apoptosis.

Infiltration of macrophages into the splenic red pulp was also a common finding and followed a similar frequency and temporal distribution as lymphoid depletion, with these two lesions being the most common findings in the spleen. Red pulp infiltration by macrophages was predominantly present at days 7, 10 and 12, only being present in one day 3 GTOV infected animal. The temporal distribution and severity were generally similar between the JUNV and GTOV infected animals and was characterized by increased number of macrophages populating the red pulp, often containing yellow-brown granular pigment (likely hemosiderin) and debris in the cytoplasm, particularly in the day 10 and 12 animals. Accumulation of fibrin within the red pulp vascular spaces as well as in the depleted marginal zones surrounding white pulp/follicles occurred in the day 12 animals (only) of both groups (Figs 2G and 5). In the JUNV infected animals it was more consistent throughout the red pulp and in the marginal zone. In the GTOV infected animals it was generally less severe and occasionally occurs in large aggregates (that may have effaced the white pulp). In three day 12 animals, including two JUNV infected and one GTOV infected, there was also mild to moderate hemorrhage in the white pulp.

*In situ* hybridization labeling of the spleen occurs on days 7, 10 and 12 and is similar in JUNV and GTOV infected animals for days 7 and 10, but consistently more severe in the day 12 animals infected with JUNV (Figs 4C and 5). Labeling is present in both the white and red pulp and strong and extensive signal often precluded localization to a specific cell type. However, labeling could specifically be identified in red pulp macrophages, vessels and red pulp vascular spaces. In a subset of animals (from both groups) the labeling was predominantly in the white pulp, including PALS, follicles and marginal zone with much less labeling in the red pulp; whereas, in others it was present in both compartments in approximately equal distribution.

**Fig 6:**
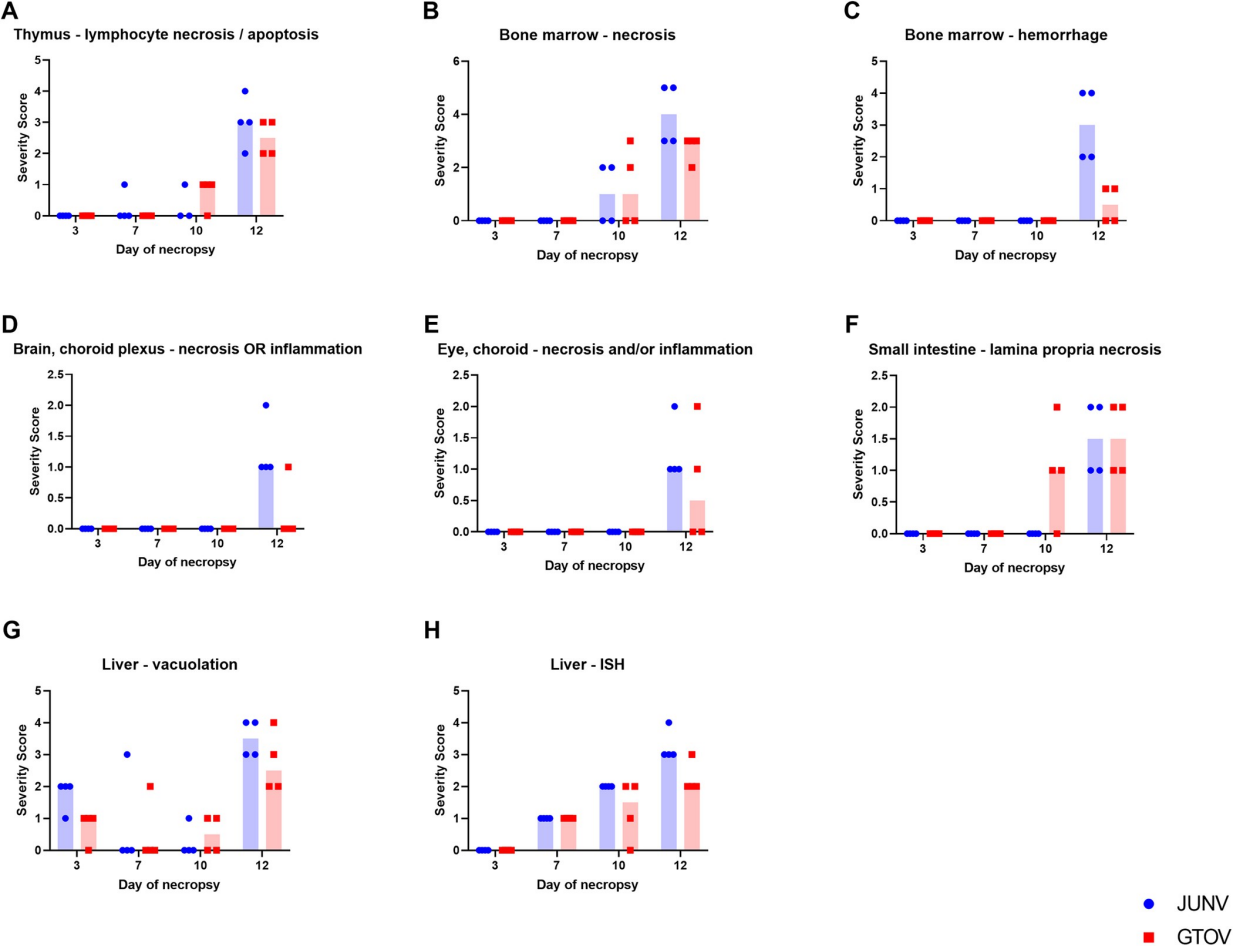
Temporal severity of histology lesions in select organs and liver ISH/viral RNA labeling (by ISH) for both JUNV and GTOV groups. In graphs A–H select histology lesions are graphed for a subset organs to display changes in lesion severity over time between JUNV and GTOV; viral RNA labeling by ISH is also graphed for the liver (graph H) to show changes in labeling severity for both viruses over time. A single dot or square represents a single animal and the blue or red shaded region represents the median.

Thymic lymphocyte necrosis/apoptosis is similar between JUNV and GTOV infected animals with mild variation in severity scores and temporal distribution (Fig 6A). This lesion was present but minimal in less than half of the day 7 and 10 animals from both groups but more severe (mild to marked) and present in all the day 12 animals from both groups. It was most predominant in the cortex and characterized by pyknotic nuclear and karyorrhectic debris, but also occurred sporadically at the corticomedullary junction and intermittently extended into the medulla, and in some cases resulted in cortical thinning and a decreased cortical: medullary ratio. *In situ* hybridization findings in the thymus includes viral RNA labeling at days 7, 10 and 12, increasing in severity over time, and following a similar temporal pattern across JUNV and GTOV infected animals with some slight variations in severity (Fig 4D). Similar to the lymph nodes and spleen, the strong and extensive signal often prevented localization to a specific cell type. However, in most samples labeling was localized to vessels/vascular tunics, stroma, capsule, thymic corpuscles and surrounding adipose tissue. Interestingly, while labeling is present in the cortex and medulla, it was often more prominent/extensive in the medulla; whereas, the majority of apoptosis was present in the cortex or near the corticomedullary (CM) junction. In general, more mature T cells are present in the medulla compared to the cortex (i.e. single positive T cells versus double positive T cells) and there are more thymic epithelial cells, dendritic cells and thymic corpuscles in the medulla [27].

Lesions in the bone marrow included necrosis, hemorrhage and fibrin accumulation. Necrosis was present at day 10 and 12 in both JUNV and GTOV infected animals but is more severe in the JUNV cohort at day 12. The lesion was characterized by abundant karyorrhectic debris and fragmented and pyknotic cells scattered throughout the bone marrow likely consisting of myeloid, erythroid, megakaryocytic and potentially stromal or other cell types such as macrophages (Fig 6B). Of note, a subset of these changes, including numerous pyknotic cells with intact membranes, were more consistent with apoptosis as seen in the lymphoid organs. However, the abundance of scattered cellular debris as well as fibrin and hemorrhage suggest a combination of both necrosis and apoptosis. The pathogenesis of this lesion may involve direct viral effects (i.e. infection followed by cell death) or may be part of a secondary process (such as ischemia, surrounding inflammation, changes in levels of cytokines and/or trophic factors). Hemorrhage and fibrin accumulation were seen in day 12 animals but were more frequent and severe in the JUNV infected animals (Fig 6C). *In situ* hybridization findings in the bone marrow included viral RNA labeling of the day 7, 10 and 12 animals for both JUNV and GTOV (Fig 4E). Temporal distribution was similar between JUNV and GTOV infected animals but there were subtle differences in severity with day 10 and 12 labeling being slightly more severe in JUNV infected animals. The majority of viral RNA labeling was multifocal but occasionally patchy or diffuse and increased over days 7–12. The signal was strong, extensive and in general was often localized to hematopoietic cells including megakaryocytes, as well as adipose tissue and vascular tunics.

### Nervous system / special senses

Nervous and special sense organs examined include the brain and eyes. Three sections of brain were examined, two transverse sections and one mid-sagittal section. The rostral transverse section included the cerebral cortex, lateral ventricles, corpus callosum, striatum and pallidum; a middle transverse section including cerebral cortex, striatum, hippocampus, thalamus, hypothalamus and third ventricle; and a caudal mid-sagittal section including cerebellum and brainstem (pons and medulla).

**Fig 7:**
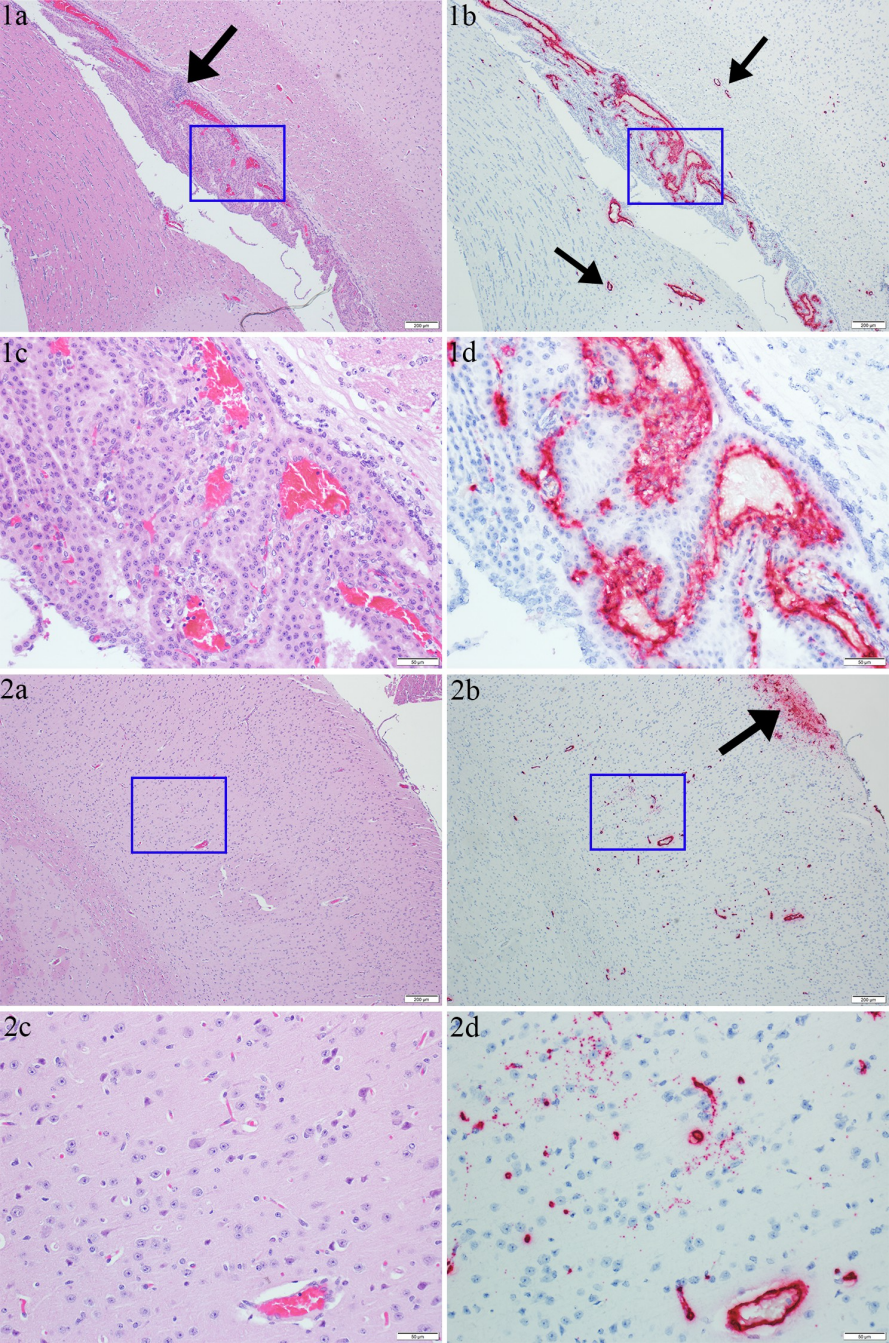
Images from the brain of a JUNV infected day 12 animal. Image group 1 represents an area of cerebral cortex at level of central hippocampus/thalamus/hypothalamus (images 1c and 1d correspond to the blue box in images 1a and 1b respectively and images 1b/d show the same area as images 1a/c). In image 1a (4x magnification H&E) at center is a lateral ventricle with choroid plexus (corpus callosum is below) and an infiltrate of lymphocytes is present in the choroid plexus (black arrow). Image 1b (4X magnification ISH; same area shown in 1a) shows an area of strong viral RNA labeling in the choroid plexus and in vessels in the adjacent neuroparenchyma (black arrows). Image 1c (20X magnification H&E) shows inflammation/necrosis in/around vessels of the choroid plexus (with endothelial hypertrophy and leakage of fibrin/edema). Image 1d (20X magnification ISH) shows strong viral RNA labeling in the center/vessels of the choroid plexus–note labeling is predominantly isolated to the center and not in the lining epithelium. Image group 2 shows an area of cerebral cortex (images 2c and 2d correspond to the blue box in images 2a and 2b respectively and images 2b/d show the same area as images 2a/c). In image 2a (4X magnification H&E) there are no appreciable histologic lesions. In image 2b (4X magnification ISH; same area shown in 2a) there is strong viral RNA labeling in vessels and in a large focal area of peripheral neuroparenchyma underlying the meninges (black arrow). In image 2c (20X magnification H&E) there are also no appreciable histologic lesions. In image 2d (20X magnification ISH; same area shown in 2c) there are areas of viral RNA labeling in the neuroparenchyma (glia, neuropil and potentially neurons) as well as strong labeling in vessels, including the endothelium.

Histologic lesions in the brain were uncommon and predominantly consisted of subtle necrosis and inflammation in the choroid plexus in the third, lateral and/or fourth ventricles and was only present in day 12 animals (Figs 6D and 7). Necrosis was only identified in a single JUNV infected animal, and inflammation was present in 75% of JUNV infected day 12 animals and 25% of GTOV infected day 12 animals (Fig 6D). Necrosis was characterized by small amounts of karyorrhectic debris in the vascular center of the choroid plexus with multifocal areas of fibrin accumulation and occasional hyalinization of capillary walls (Fig 7, image 1c). Inflammation was characterized by accumulation of lymphocytes and macrophages in the vascular center of the choroid plexus with accumulation of fibrin and/or edema around or within the capillary walls. These lesions were likely associated with the presence of viral RNA or virus in the choroid plexus (see below). The nature and location of viral labeling by ISH in many tissues is consistent with some degree of vascular tropism. Viral RNA labeling was present at days 10 and 12, was approximately similar between JUNV and GTOV infected animals and generally increased in severity from days 10 to 12 (Figs 4F and 7). Viral RNA labeling was present throughout all three sections of brain and the majority of labeling was noted in vessels of the meninges, neuropil (predominantly gray matter) and choroid plexus. Significant labeling was not noted in the epithelial lining of the choroid plexus, but rather is concentrated in the center, which is capillary rich with a thin collagen matrix. In the majority of animals there was at least some degree (either minimal focal or multifocal) of labeling of neuroparenchymal (neuropil, neurons, and/or glia) components but was less common than labeling of adjacent vessels (Fig 7, images 2b and 2d).

**Fig 8:**
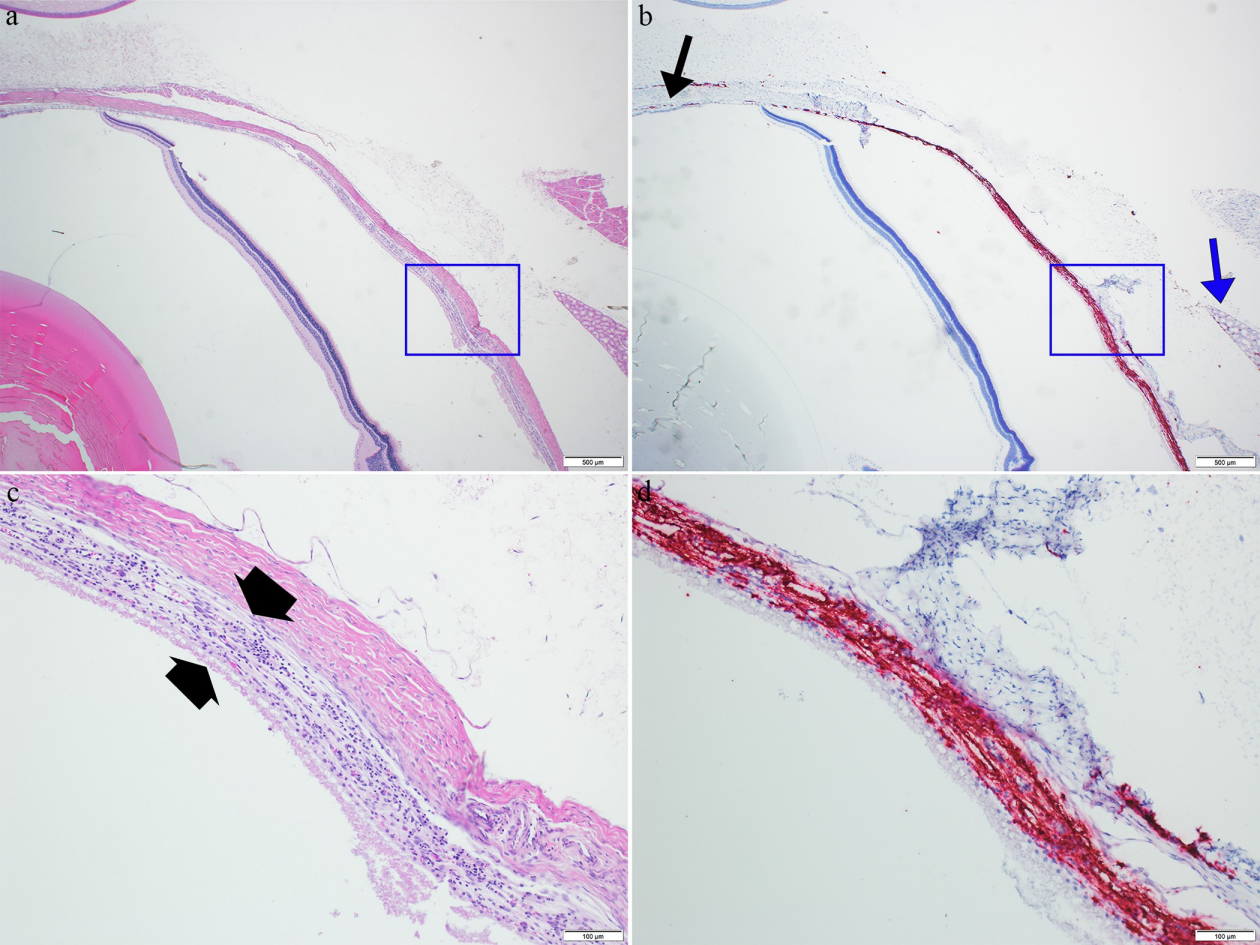
Images from the eye of a JUNV infected day 12 animal. Images a and b are from the same region and images c and d represent blue boxes in images a and b respectively. Image a (4X magnification, H&E) shows a thickened choroid; retina is artificially detached from choroid. In image b (4X magnification, ISH) there is strong viral RNA labeling (red) in choroid extending anterior toward the anterior uvea (black arrow); note also the small section of lacrimal gland with a minimal amount of labeling (blue arrow); part of the sclera is artifactually separated from the choroid. Image c (20X magnification, H&E) shows the choroid (between thick black arrows) thickened with infiltrating mononuclear inflammatory cells; note the retina is artifactually separated with only thin layer of outer retina remaining attached. Image d (20X magnification, ISH) shows strong viral RNA labeling in the inflamed, thickened choroid; note the sclera has partially, artifactually separated from the choroid).

Histologic lesions in the eye most likely associated with viral infection include minimal or mild inflammation and necrosis in the choroid and necrosis in the conjunctival lymphoid tissue (Figs 6E and 8C). These lesions were seen exclusively at day 12 and predominantly in the JUNV infected group. Two (50%) day 12 JUNV infected animals had minimal to mild apoptosis/necrosis of lymphoid tissue in the conjunctiva, one of which also had hyperplastic lymphoid tissue. The lesion likely has the same origin/pathogenesis as the similar lesion seen in the lymph nodes. Both animals had viral RNA labeling in the lymphoid tissue, similar to what is described in the lymph nodes which provides further support for viral predilection for lymphoid tissue. There is also viral RNA labeling in the adjacent conjunctival epithelium. The two lesions in the choroid of the eye are reminiscent of the lesions seen in the choroid plexus of the brain. Inflammation was characterized by either infiltration of lymphocytes and/or macrophages, or rarely granulocytes (eosinophils or neutrophils) into the choroid in low numbers with thickening/expansion of the choroid (Fig 8). The necrosis is characterized by very small amounts of karyorrhectic debris in the thickened choroid. Inflammation and/or necrosis was seen in 100% of the day 12 animals in the JUNV-infected group, but only 50% of the day 12 animals in the GTOV group (Fig 6E).

*In situ* hybridization viral RNA labeling was seen on days 7, 10 and 12 in both JUNV and GTOV-infected animals but predominantly on days 10 and 12 with an increase in severity on day 12 (Figs 4G and 8). Labeling was seen in a similar temporal pattern between JUNV and GTOV infected animals, but day 12 animals in the JUNV group had consistently higher severity scores (Fig 4G). Labeling was frequently bilateral and noted in the choroid, anterior uvea (iris and ciliary body), sclera/cornea at the limbus, conjunctiva including epithelium and lymphoid tissue. Labeling was not seen in all locations in each animal but was seen in most locations in the most severely affected animals and the choroid was one of the most consistent locations for labeling where it was often strong and extensive (Fig 8). In the anterior uvea labeling was seen in the connective tissue core/stroma and not the epithelium. In some of the more severely affected JUNV animals labeling was also present in the connective tissue and lacrimal glands adjacent to the eye. The pattern of labeling in the eye may also reflect vascular viral tropism as labeling was seen in areas with numerous capillaries (i.e. scleral/corneal limbus and choroid) and in vessels. Interestingly, a low number of animals in all groups, including one control, had conjunctival lymphoid hyperplasia and three of the either JUNV or GTOV infected animals (one day 7, two day 12) also had viral RNA labeling in the hyperplastic lymphoid tissue. It is unclear whether the virus resulted in lymphoid hyperplasia or whether it was trafficked into the lymphoid tissue as part of antigen presentation, particularly given the potential predilection of the virus for lymphoid tissue as seen in the lymph nodes, spleen and thymus. A single day 7 animal in the JUNV-infected group had a focal mononuclear infiltrate in the posterior sclera adjacent to the optic nerve head, localized to the connective tissue adjacent to the optic nerve. It is likely related to viral infection as there is viral RNA labeling localized to the infiltrate and surrounding the optic nerve. Both labeling and lesion severity were minimal in degree but significant given the likely causal association, early presence (i.e. day 7) and location near the optic nerve.

### Cardiopulmonary system

**Fig 9:**
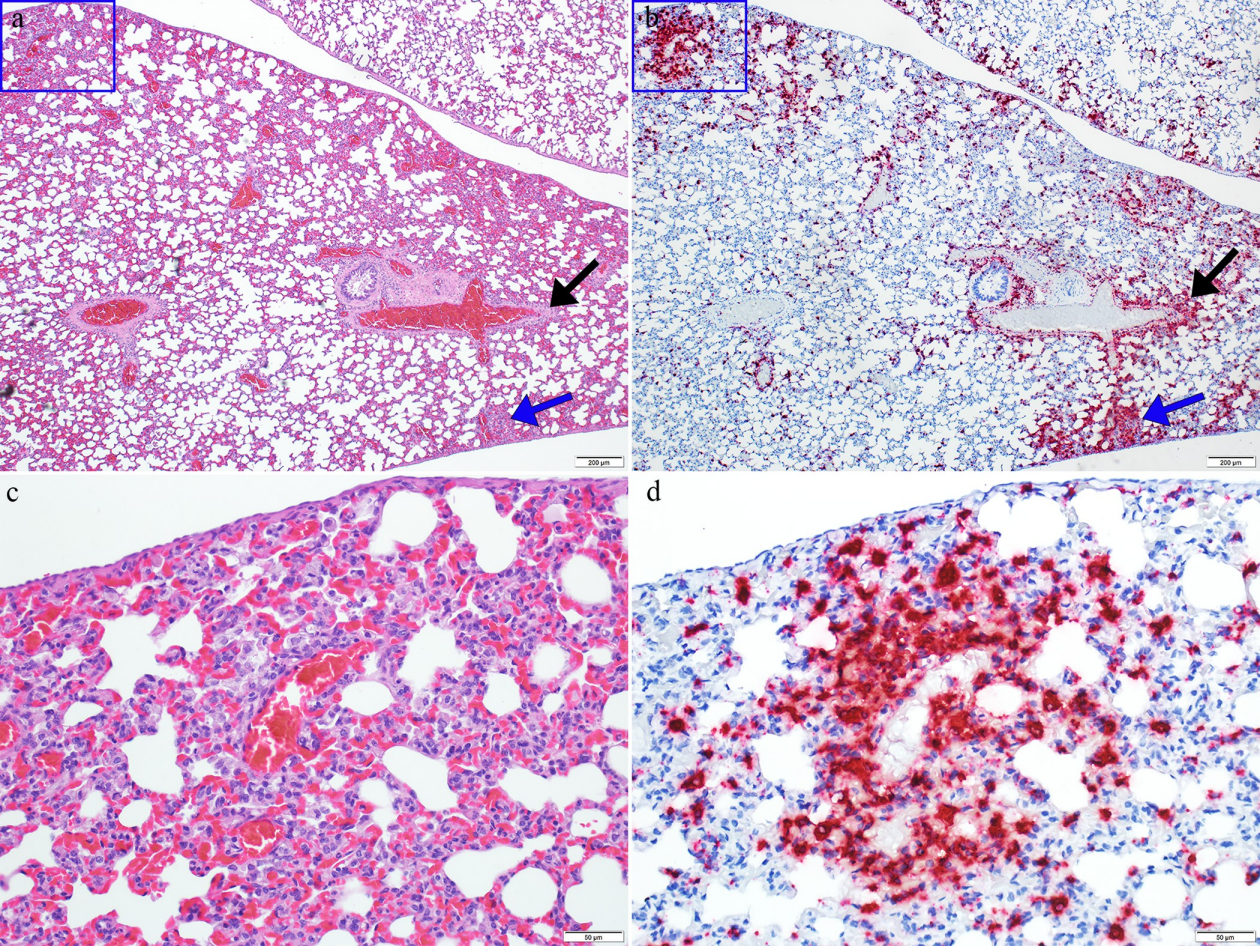
Images from the lung of a GTOV infected day 12 animal. Images a and b are from the same region; images c and d represent blue boxes in images a and b respectively. In image a (4X magnification, H&E) there are multifocal areas of minimal alveolar inflammation (blue arrow and blue box) that are often associated with small vessels, and areas of mononuclear infiltrate surrounding larger vessels (black arrow). In image b (4X magnification, ISH) there is strong viral RNA labeling in areas of alveolar inflammation and labeling in mononuclear infiltrates surrounding vessels. In image c (20X magnification, H&E) there is minimal mononuclear alveolar inflammation, at the center of which is a small vessel surrounded by a mononuclear infiltrate (perivascular). Image d (20X magnification, ISH) is the corresponding ISH image and shows strong viral RNA labeling in the area of alveolar inflammation and surrounding the small vessel at center.

Lesions in the lung were relatively uncommon and when present either of minimal or mild severity. Most lung lesions fall into the equivocal category, meaning they may or may not be directly related to arenavirus infection. Additionally, some of the lesions such as perivascular infiltrates (i.e. infiltration of low numbers of inflammatory cells in the absence of other features of inflammation) were noted in the control animals and are known to be a background finding in guinea pigs and of unknown etiology [28]. Perivascular infiltrates of mononuclear cells, predominantly lymphocytes, and perivascular inflammation were seen collectively as the most common lung lesions and occurred as small nodules adjacent to or surrounding small to medium sized pulmonary vessels (Fig 9A). Viral RNA labeling is associated with some, but not all, of these perivascular lesions. Perivascular inflammation was present in four JUNV animals, from days 7, 10, 12. Perivascular infiltrates were seen in the majority of animals in the study, including three control animals, and at all time points for both JUNV and GTOV with the exception of day 3 JUNV infected animals. Given the distribution of viral RNA labeling, these infiltrates may be associated with infection, but are considered equivocal given their presence in control animals and as a documented background lesion of guinea pigs.

The remaining lung lesions were rare and in most cases of minimal severity. Minimal alveolar inflammation was present in nine animals including one control. The nature of the inflammation was multifocal and variable either being composed of mononuclear cells (lymphocytes and/or histiocytes) and heterophils or eosinophils within alveolar lumina or expanding alveolar septa (Fig 9C). This inflammation is more consistently present in the JUNV infected animals and the temporal distribution (i.e. more consistent in days 10 and 12) suggests these lesions may be associated with viral infection. Additionally, in some cases there is more pronounced viral RNA labeling in areas with alveolar inflammation also suggesting the lesion is secondary to viral infection (Fig 9). Bronchiolar lesions included rare minimal to mild infiltrates of mononuclear inflammatory cells, with fewer heterophils or eosinophils, and are present in both groups at multiple time points but were more common in GTOV infected animals. The infiltrates were often concentrated around bronchioles in the adventitia but also extended into the muscular layer and occasionally to the lamina propria. This finding was relatively rare, only found in five animals. Viral RNA labeling was frequently noted associated with the infiltrates suggesting they may be related to infection.

Viral RNA labeling in the lung is discussed above in reference to specific lesions, but was also noted in other areas throughout the lung and ranged in severity from minimal to marked (Fig 4H). Severity increased over time with marked labeling present in day 12 JUNV infected animals (Fig 4H). RNA labeling was identified in the alveolar septa (pneumocytes, macrophages, capillaries); infiltrates and areas of inflammation as previously discussed; small vessels (endothelium, tunica media, tunica externa); adventitia and smooth muscle of bronchioles; bronchus-associated lymphoid tissue (BALT); and occasionally mesothelium (Fig 9). Similar to other tissues examined in this study, labeling was often noted in the absence of appreciable histologic lesions (Fig 9). Temporal labeling was similar and increased over time for both JUNV and GTOV infected animals with the day 12 JUNV infected animals receiving a slightly higher severity score (Fig 4H). The extensive labeling suggests the lung may be a target tissue for this virus, potentially due to the pulmonary vascular pattern, but also indicates that in many cases the response to viral infection, even in the later time points, is relatively subdued.

The primary lesion in the trachea was an infiltrate in the lamina propria and mucosa composed of eosinophils or heterophils in the epithelium, lamina propria and occasionally extending to the submucosa with no obvious tissue damage. The lesion was seen on days 3, 7 and 10 in both GTOV and JUNV infected animals with no relationship to time point, and was also present in control animals suggesting this may be an equivocal lesion (i.e. may or may not be related to infection). There is viral ISH labeling which increases between days 10 and 12 for both JUNV and GTOV infected animals. Labeling was predominantly in the submucosa and lamina propria including submucosal vessels as well as the mucosal epithelium, particularly in the more severely affected animals.

Lesions in the heart were very rare and included minimal mononuclear and/or heterophilic inflammation seen in the atrial septa in a day 3 JUNV infected animal and also in a control, indicating this lesion is most likely incidental, supported by a lack of corresponding ISH labeling. Two day 12 animals, one in each group (JUNV and GTOV), had minimal mononuclear inflammation in the endocardium. In both animals the lesion was very small and focal or multifocal, both in the right ventricle and both with viral RNA labeling in the lesion. Rare, unattached fibrin thrombi are seen in the ventricles or atria of two JUNV infected animals (days 10 and 12) that may have occurred as a consequence of infection or endothelial damage, but may also be incidental given their rarity and minimal degree. Viral RNA labeling in the heart was noted in both day 10 and 12 JUNV and GTOV infected animals which increases from day 10 to day 12 in a similar pattern between groups. Labeling was present most consistently in the endocardium and myocardial vessels, but also in cardiac myocytes, most often in a nuclear or perinuclear location. Labeling was seen in most regions of the heart including both ventricles, the interventricular septum, atria, auricles and papillary muscles.

### Hepatobiliary system

The majority of significant lesions in the liver were equivocal. Additionally, there was an extensive set of background or incidental lesions in the liver which have been documented in guinea pigs and which will be briefly discussed at the end of this section. Hepatocellular vacuolation, both microcytic and macrocytic, was the most consistent finding likely related to viral infection, although it was also present to a minimal degree in the control animals (Fig 6G). In the control animals and the majority of infected animals in either group the lesion was macrocytic (i.e. larger sized, often single, clear vacuoles in the hepatocyte cytoplasm that may compress the nucleus). Fewer infected animals showed a microcytic hepatocyte vacuolation pattern (i.e. many smaller vacuoles filling the cytoplasm). These animals were generally in the day 10 or 12 cohorts for both JUNV and GTOV, suggesting viral infection likely played a role in the lesion, either directly or indirectly (Fig 6G). The patterns of vacuolation were either diffuse or peripheral (i.e. at the periphery of lobes) and variably present in all zones (i.e. centrilobular to midzonal or periportal to midzonal).

The remaining lesions were much less common and occurred somewhat sporadically in both groups. Portal necrosis was seen in one day 12 animal in the JUNV group and was characterized by small amounts of apoptotic/necrotic debris in the portal region, likely representing individual cell necrosis of biliary epithelium, lymphocytes or endothelial cells. Periportal inflammation or inflammation consisting of predominantly mononuclear cells was seen in a low number of both JUNV- and GTOV-infected animals. There was not a consistent pattern between groups (i.e. it occurred in different day cohorts in JUNV- versus GOTV-infected animals) but the lesion was not seen in control groups and therefore may be related to infection. An adherent fibrin thrombus was seen in the central vein in a single day 10 JUNV infected animal which may have occurred as a consequence of endothelial infection and/or damage. Viral RNA labeling in the liver is seen on days 7, 10 and 12 in both JUNV and GTOV infected animals, and increases from days 7 to 10/12 (Fig 6H). Labeling was present in hepatocytes; sinusoids (Kupffer cells, other sinusoidal lining cells); portal areas (endothelium, other); the periphery of areas of necrosis (degenerate hepatocytes, biliary hyperplasia–note this is the background lesion discussed below). In the animals with higher scores (i.e. day 12) there is a slight zonal pattern with more labeling around central veins and portal regions in some areas, which is similar for both JUNV and GTOV.

There was a constellation of lesions in the liver of multiple animals in both groups as well as the control animals that have been documented as background or incidental lesions in guinea pigs [28], but will be described briefly due to their extensive nature. These lesions include hepatocellular necrosis and degeneration with hepatocyte loss, biliary epithelium hyperplasia, hemorrhage, fibrosis and mineralization. The precise cause of these lesions is unclear. These lesions were seen in both groups, as well as the control animals, at multiple time points and with no clear pattern of occurrence. Additionally, this constellation of lesions was seen in animals with no viral labeling and in animals with increased viral labeling in both JUNV and GTOV infected animals; however, labeling was not seen in necrotic hepatocytes. While the reason for this increased labeling is unclear, it is postulated to be related to changes in vascular patterns associated with these incidental lesions, followed by increased viral RNA in the area due to the proclivity of the virus for blood vessels, resulting in increased labeling. These lesions have been previously described/discussed and similar findings are reported elsewhere in the literature [28–30].

Lesions in the gallbladder were noted in one animal with a mononuclear infiltrate. There was viral RNA labeling in a temporal pattern similar to that observed in other organs which was most severe at day 12 in both JUNV- and GTOV-infected animals. Labeling was predominantly in the submucosa and lamina propria, along the basement membrane, in vessels and lymphatics and rarely in the epithelium on the animals with higher labeling scores. JUNV animals showed consistently higher labeling scores than GTOV infected animals.

### Digestive system

The pancreas and salivary gland will be discussed separately, and the remaining gastrointestinal organs will be discussed collectively as a group including esophagus; stomach; duodenum; jejunum; ileum; cecum; and large intestine. Histologic lesions in the pancreas were rare and included minimal inflammation in the peripancreatic adipose tissue noted in two JUNV and one GOTV animal with no specific temporal pattern. There was viral RNA labeling in the pancreas in both groups at days 10 and 12, with the majority in the exocrine acini, the interstitium, or rarely in islets of Langerhans. There were also foci of labeling in the adjacent peripancreatic adipose tissue / mesentery in several cases. Labeling in day 12 JUNV-infected animals had a consistently higher score compared to labeling in day 12 GTOV-infected animals.

Salivary gland lesions included periductal mononuclear inflammation seen in the mixed/mandibular salivary gland of one day 10 JUNV-infected animal and one day 12 GTOV-infected animal. The inflammation was associated with enlarged ductal epithelial cells with large, eosinophilic, 2–4μm, intranuclear inclusion bodies. This lesion is characteristic of guinea pig cytomegalovirus [28], present as a latent or persistent infection in many guinea pigs. Although it may be considered an incidental finding, it may also suggest some degree of immunosuppression in these animals. The agent was confirmed with ISH viral RNA labeling which was multifocally positive in rare ductal epithelial cells. Viral RNA labeling for arenavirus was also identified in the salivary gland interstitium, and rarely in vessels and epithelium in days 10 and 12 JUNV and GTOV infected animals and did not correlate with histologic lesions.

**Fig 10:**
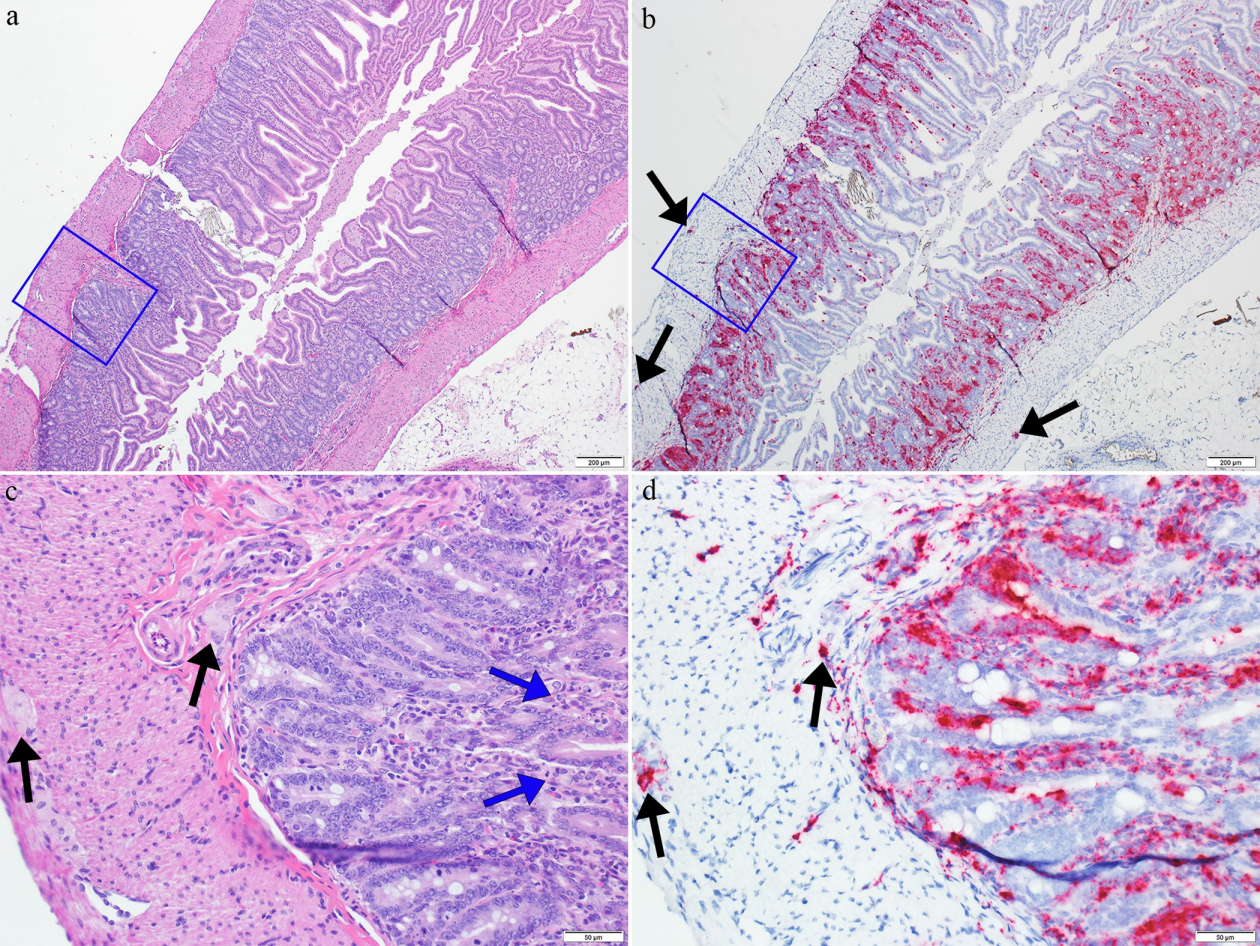
Images from the small intestine (jejunum or ileum) of a GTOV infected day 12 animal. Images a and b are from the same region; images c and d represent blue boxes in images a and b respectively. In image a (4X magnification, H&E) there is minimal dilation of villar lamina propria with small amounts of apoptotic debris. In image b (4X magnification, ISH), the corresponding ISH images there is strong viral RNA labeling in the lamina propria and submucosa with occasional labeling in the muscular layers; there is prominent labeling in intestinal nerve plexi (submucosal and myenteric) (black arrows). In image c (20X magnification, H&E) there are small amounts of apoptotic debris in the lamina propria (blue arrows); black arrows indicate submucosal and myenteric nerve plexi. In image d (20X magnification, ISH), the corresponding ISH image, there is strong viral RNA labeling in the lamina propria, extending into the submucosa; note rare labeling in the inner muscle layer and labeling in nerve plexi (black arrows).

Esophageal and gastric lesions did not follow a specific pattern or correlate with viral RNA labeling. The most consistent lesion in the small and large intestine consisted of necrosis/apoptosis in the lamina propria characterized by small amounts of apoptotic or karyorrhectic debris in the lamina propria, often in or around mildly dilated lymphatics (Fig 10C). The necrosis or apoptosis may represent remnants of infiltrating mononuclear inflammatory cells (i.e. macrophages or lymphocytes) and/or endothelium lining vessel and/or lymphatics. The lesion was present predominantly at day 12 in the small and large intestines and was similar between GTOV and JUNV infected animals with the exception of being present at day 10 in GTOV infected animals but not at day 10 in JUNV infected animals (Fig 6F). Other lesions in the intestine included hyperplasia of the gut associated lymphoid tissue (GALT) and apoptosis/necrosis of the lymphoid tissue in both JUNV and GTOV infected animals. Minimal hyperplasia of the GALT was also observed in control animals. GALT hyperplasia was most commonly seen in the ileum and most consistent in day 3 and 7 GTOV infected animals. Apoptosis/necrosis in the GALT was a rare finding in the day 10 and/or day 12 animals in both groups. It is presumed to have a similar pathogenesis to the lesions described above for other lymphoid tissues. Viral RNA labeling was present in both hyperplastic lymphoid tissue as well as areas with apoptosis and is discussed next (Fig 10).

Viral RNA labeling was seen on days 10 and 12 in the esophagus and stomach and in days 7, 10 and 12 in the esophagus, stomach and small intestine (Fig 4I). ISH was not performed in the large intestine/cecum. The labeling patterns (temporal and spatial) between JUNV- and GTOV-infected animals were similar but the severity scores in the JUNV day 12 animals were higher than those of the GTOV-infected animals (Fig 4I). Gastrointestinal organ labeling was predominantly in the lamina propria including vessels but also extended into the submucosa. Labeling was also present in a focal or multifocal, often sporadic pattern between or within muscle layers and in vessels, including the adventitia (Fig 10). In the esophagus there were multifocal, patchy, but prominent areas of labeling of the squamous mucosal epithelium. The highest amount of labeling occurred in the small intestine and was often seen extending into the villar epithelium (Fig 10). Interestingly, specific, strong and consistent labeling was also present in the myenteric and submucosal plexi (in both groups) in the small intestine, suggesting this agent may also target the enteric nervous system (Fig 10). This labeling was also present in the stomach, but was less prominent. The overall labeling pattern shows an increase over time from day 7 to 12 (Fig 4I).

### Endocrine system

Endocrine organs examined include the adrenal gland and thyroid gland. Lesions were rare in the adrenal gland and include both equivocal lesions and lesions most likely related to viral infection. Equivocal lesions included rare, minimal inflammatory infiltrates in either the periadrenal adipose tissue, cortex or medulla. Infiltrates in periadrenal adipose tissue were rarely located within or around the large ganglion positioned adjacent the adrenal gland. Lesions were generally either mononuclear and/or heterophilic and occurred sporadically in both groups but were more common in JUNV infected animals. Other equivocal findings in the adrenal gland included cytoplasmic vacuolation and mineralization in the cortex. Cytoplasmic vacuolation was noted in nearly all animals in the study including controls; however, the vacuolation discussed here was of greater incidence and/or severity than noted in the controls. The lesion was present in a day 7 and a day 12 GTOV infected animal and was isolated to the zona fasciculata. It was characterized by marked expansion of the cytoplasm with compression of the nucleus (often completely obscured) and cell swelling. It is presumed to represent a degenerative change but its relationship to viral infection is unclear due to the rarity of the lesion. Mineralization was noted in the cortex (zona fasciculata and/or reticularis) in three day 12, GTOV infected animals and was presumed to be dystrophic and potentially related to degeneration or necrosis in the cortex, although its rarity makes it difficult to draw specific conclusions about the origin. The remaining lesions in the adrenal cortex were also rare and most likely directly related to viral infection. These lesions include degeneration and/or necrosis, hemorrhage, and inflammation. The degeneration and/or necrosis was characterized by either cell swelling with vacuolation and/or shrunken hypereosinophilic cells with rare nuclear pyknosis, karyorrhexis and fragmentation (and mineralization in one day 12 GTOV infected animal) in the zona fasciculata with and without hemorrhage. Inflammation was seen in three day 12 animals (one JUNV, two GTOV) and was composed of either heterophils or macrophages and lymphocytes. Hemorrhage was a consistent lesion seen only in the four day 12 JUNV infected animals and surrounded and separated adrenal cortical cells in the zona fasciculata and reticularis in either a focally extensive or multifocal pattern. In general the lesions in the adrenal gland were significant based on their likely relationship to viral infection; however, their relative infrequency and low severity scores make drawing specific conclusions about the lesions in this organ problematic.

Viral RNA labeling in the adrenal gland was seen predominantly in day 10 and 12 animals, with increasing severity on day 12, but was also seen in one day 7 JUNV animal and two day 7 GTOV animals and was minimal in all three. In day 10 animals labeling ranged from minimal to mild in both groups. In day 12 it ranged from moderate to marked in JUNV infected animals and mild to moderate in GTOV infected animals. Multifocal labeling was present in all layers of the adrenal cortex as well as in the adrenal medulla and adrenal capsule including the cortical epithelium, medullary cells and in vessels. Labeling was occasionally noted multifocally in the areas of degeneration/necrosis described above, but not all areas of degeneration and necrosis had labeling present. Interestingly, in sections of adrenal gland with adjacent ganglia present in day 10 and 12 animals, there was occasional labeling in ganglion neurons, particularly in the day 12 animals.

Histologic lesions in the thyroid gland were not observed in this study. However, viral RNA labeling was observed in the thyroid gland on days 10 and 12 in both JUNV- and GTOV-infected animals sharing a similar pattern in the interstitium (between follicles), which may include labeling of endothelium and/or parafollicular/C cells, and rare follicular cells.

### Urinary system

Urinary system organs examined included the kidney and urinary bladder. Lesions in the kidney consisted of inflammatory infiltrates, predominantly mononuclear, in the renal pelvis, cortical and medullary and, rarely, perivascular interstitium. This lesion was frequent at all time points in both JUNV and GTOV infected animals, with a slightly higher frequency on day 10 and 12, and was also seen in one control animal. The renal pelvis was the most common location for the infiltrates. Lesions were not noted in the urinary bladder.

Viral RNA labeling was present in the kidney and urinary bladder in the majority of day 10 and 12 animals from both groups. In day 10 animals from both groups the kidney and urinary bladder labeling was minimal. In the day 12, JUNV-infected animals labeling ranged from minimal to moderate in the kidney and was mild in the urinary bladder. In the day 12 GTOV-infected animals, labeling in the kidney was minimal and was minimal to mild in the urinary bladder. In both groups labeling in the kidney was predominantly in the interstitium of the cortex and medulla including vessels, lamina propria underlying the urothelium of the renal pelvis, and rarely the urothelium of the day 12 animals. Labeling often accompanied inflammatory infiltrates in the renal pelvis. In the urinary bladder, labeling was predominantly in the lamina propria underlying the urothelium, but was also rarely seen in the urothelium as well as tunica muscularis and vessels (day 12 animals).

### Reproductive system

Reproductive organs examined include the ovary and uterus. Lesions in the ovary were rare, present only on days 10 and 12 and included mononuclear inflammation or infiltrates at various locations in the ovary, occasionally associated with small vessels, in both GTOV and JUNV animals. Minimal apoptosis/necrosis was also identified in the stroma of one day 12 JUNV infected animal. Similar lesions were noted in the uterus and included mononuclear inflammatory infiltrates in the myometrium or endometrium that were occasionally perivascular. The overall character and temporal occurrence of these lesions suggests they may be associated with viral infection; however, the minimal severity and overall rarity of lesions suggests days 10 and 12 may be relatively early in the infection process or these organs are minimally targeted.

Viral RNA labeling was present in the ovaries and uterus on days 7, 10 and 12. In day 7, JUNV labeling was minimal and only present in 1 animal each in both the uterus and ovaries, similar to what was noted in GTOV-infected animals. All day 10 and 12 animals from both groups had labeling present. In day 10 animals from both groups the severity ranged from minimal to mild in both the uterus and ovaries. On day 12, JUNV labeling ranged from mild to moderate in the ovaries, and minimal to moderate in the uterus. On day 12, GTOV labeling ranged from mild to marked in the uterus and mild to moderate in the ovaries. In both groups, labeling in the in the ovary was present prominently in the thecal layer surrounding follicles, as well as in the corpora lutea and stroma, including vessels and in atretic follicles. In the uterus labeling was noted in the endometrial stroma including vessels, myometrium and limited labeling in the endometrial epithelium in a subset of day 12 animals.

### Musculoskeletal system

Sections of skeletal muscle were examined and no histologic lesions were found in either group. Viral RNA labeling was present on days 10 and 12 in both JUNV and GTOV infected animals and was localized to the endomysium, occasionally perinuclear in myocytes, in the perimysium/interstitium and around vessels. This pattern was similar to what was seen in other organs with labeling in the absence of a lesion, with and a proclivity for interstitial tissues. Other incidental histologic findings were recorded in various organs but are considered background lesions unrelated to the study and will not be discussed further.

## Discussion

The most severely targeted organs in terms of lesions and viral load were the hemoatolymphoid organs including the lymph nodes, thymus, spleen and bone marrow (and to a lesser extent the gastrointestinal lymphoid tissue). The mesenteric lymph node was the first site where viral RNA was detected, in a single day 3 JUNV infected animal, consistent with the postulated pathogenesis which includes infection of monocyte/macrophage lineage cells in the tissue initially exposed to the virus, followed by infection of the local lymphoid tissue. The pattern of histologic change in these organs suggests there may be early lymphoid hyperplasia (cortical and/or paracortical) with histiocyte infiltration, followed by lymphoid depletion at later time points. It is unclear if the changes are due to direct (primary) viral infection of hematopoietic and lymphoid cells, dendritic cells and macrophages with failure of activation resulting in apoptosis/necrosis and lymphoid depletion [2,3]; or indirect (secondary) to generalized immunosuppression, lymphoid exhaustion [2] and/or inflammation in affected organs. Viral antigen has not been identified within lymphocytes (in human reports) suggesting a more intricate mechanism than primary viral infection induced death as being responsible for the observed lymphocyte apoptosis/necrosis and depletion [16].

The precise pathogenesis of decreased lymphocyte density in this study is likely complex and multifactorial. This is particularly true given the lesions in the thymus and bone marrow at the later time points (i.e. day 12), the abundance of viral RNA present in the lymph nodes, and the presence of lymphoid apoptosis/necrosis at the later time points (i.e. days 10 and 12). Decreased lymphocyte cellularity may be due to increased cell death (evidenced by apoptosis/necrosis) and/or decreased production related to changes in the bone marrow and/or thymus, or perhaps both. Additionally, viral RNA in the lymph nodes may affect lymphocytes and/or fibroblastic reticular cells (FRCs)/stromal cells thereby influencing lymphocyte trafficking and distribution. Viral antigen has been described in fibroblastic reticular cells in lymph nodes with experimental infection of guinea pigs with Lassa virus [31]. Increased lymphocyte apoptosis can also occur as a consequence of viral infection, either as a result of stress (glucocorticoid release) or direct viral effects. Increased lymphocyte apoptosis in the thymus for example is often a consequence of stress, such as from viral infection. Given the high levels of viral RNA in the lymph nodes, direct viral effects are also plausible and further investigation of those mechanisms is warranted. Direct effects may include infection of lymphocytes, secondary effects on lymphocytes from infection of stromal cells such as fibroblastic reticular cells [32] and/or macrophages and tissue specific changes in levels of cytokines or trophic factors. Ongoing increased apoptosis/necrosis in this case likely contributed to the lymphocyte depletion as discussed above. Viral infection in general can cause a non-specific decrease in lymphocyte cellularity and because multiple lymphoid organs are affected in this study (i.e. thymus, spleen, and lymph nodes) suggests a systemic deleterious immunomodulatory effect may play a role. The identification of recrudescent, latent guinea pig cytomegalovirus in at least two animals (one in each viral group) in this study supports the concept of a global immunosuppressive environment playing an important role in this infection. Additional investigations to identify specific cell types infected (e.g. duplex immunofluorescence assay and/or electron microscopy) and tissue specific profiles of immunosuppressive cytokines and/or immunoregulatory cytokines such as type I interferons, should aid in understanding the depletion of lymphoid tissues and immunosuppressive aspects of this infection [2].

Immunosuppression is an important element of arenavirus infection and the ability of the immune response to control the level of viremia early in NW arenavirus infection is an important factor influencing survival [3]. The high viral loads which may result in T cell exhaustion are likely an important component of the immunosuppressive mechanisms of arenavirus infection [2]. Monocytic cells act as an early target of infection but apparently do not result in increased cytokine production/activation as demonstrated for JUNV infection [33]. It is postulated they may act as a reservoir for infection and spread the virus to draining lymph nodes [3]. Alterations in the immune response induced by arenavirus infection are complex and vary between OW and NW arenaviruses. Influencing cytokine profiles, such as interferon (IFN) expression levels, through viral protein interaction (i.e. nucleoproteins and/or Z protein) with various components of the IFN pathway appears to be an important component of immunosuppression [2,11,34]. For example it has been shown that in fatal human cases of AHF, IFN-α levels were much higher than in survivors [35]. Indeed, we also observed high levels of serum IFN-α that corresponded to viral load. Antagonism of the IFN pathway by a myriad of mechanisms is a common strategy employed by many virus families, including those containing other hemorrhagic fever causing viruses, to interfere with the host immune response [36,37]. AHF patients also have increased levels of cytokines including IL-6, IL-8, IL-10 and TNF-α that are associated with disease severity [38]. A more thorough understanding of the mechanism(s) by which NW arenaviruses may circumvent viral inhibition by the IFN pathway and result in immunosuppression will aid in understanding the disease pathogenesis and may also guide the development of therapeutic targets [34]. Correlating the pathology findings with viremia data and cytokine analysis is crucial to understanding the immunosuppressive aspects of NW arenavirus infection. Comparison of these findings across all the pathogenic NW arenaviruses will advance our understanding of the potential for cross protection of vaccines and therapeutics.

Other published studies with JUNV in guinea pigs showed similar findings in the spleen and liver to what was reported here [21]. Additionally, GTOV administered subcutaneously in guinea pigs has also been reported to cause lung lesions (interstitial pneumonia and hemorrhage) at days 12–14 [20]. These previous studies in combination with ours confirms the consistency of findings in the Guinea pig model of NW arenavirus infection and simultaneously reveals the absence of complete data, including all organ system pathology and virus distribution, in this model, which we provide in this manuscript.

Vascular involvement, including the endothelium, and in other layers of larger vessels (i.e. tunica media and externa) appears to be an important aspect of these infections. The robust labeling signal from the RNAscope ISH procedure in many cases prevented localization of the signal to a specific cell type (i.e. endothelium of small capillaries). Nonetheless, viral RNA labeling was often intense in the stromal elements and interstitium of tissues, where vessels are more concentrated, as opposed to cells of the parenchyma. Labeling was occasionally seen in the epithelium of organs and tissues such as the esophagus, small intestine, salivary glands and conjunctiva. However, labeling was much less common than in the interstitium and stroma of nearly all organs. Labeling was especially prominent in the vessels of the brain. Additionally, prominent labeling was also present in the choroid layer of the eyes along with histological changes such as inflammation and necrosis, similar to the changes seen in the choroid plexus of the brain. The choroid layer of the eye is highly vascular with numerous capillaries in a thin collagen and elastin matrix which may suggest these changes are part of virus mediated vascular damage. Infection of endothelium in the absence of cytopathic effect has been previously documented [39] and this study is supportive of that finding for both JUNV and GTOV. Infection of endothelial cells likely plays an important role in disease pathogenesis and while direct cytopathic effect is not observed, alterations in cell function, such as changes in adhesion molecule expression, coagulation factor release and nitric oxide (NO) and prostaglandin production may be affected and drive the disease process [11,39,40]. Thrombocytopenia is also a component of the clinical syndrome of AHF. JUNV has been found to replicate in megakaryocytes and it has been postulated that the observed decrease in thrombopoiesis may be related to elevated levels of type I interferon [14]. A complex set of events involving endothelial infection and subsequent dysfunction in combination with megakaryocyte infection and thrombocytopenia likely create a disordered coagulation microenvironment leading to the hemostatic abnormalities and bleeding tendencies associated with hemorrhagic fever secondary to NW arenavirus infection [14]. The splenic and bone marrow fibrin accumulation observed in this study also support the presence of endothelial dysfunction and/or damage as the pathogenesis is related to either damage to vascular endothelium, release of inflammatory mediators and/or changes in blood flow dynamics (stasis), or some combination thereof. Further studies should focus on NW arenavirus disruption of endothelial homeostasis within the infected host to illuminate key features of pathogenesis.

Another interesting pattern that emerged from analysis of the histology findings combined with the ISH data is the repeated finding of viral RNA labeling in the absence of significant histologic lesions (i.e. inflammation and/or necrosis), or lesions with minimal to mild severity scores. Often when histologic lesions were present (e.g. in the heart or lung), there were of limited severity, but did correlate with viral RNA labeling. Generally, these rare lesions, when present, consisted of minimal to mild areas of mononuclear (lymphocytes and/or macrophages) infiltration or inflammation. These observations support an immunosuppressive and/or immunoevasive component to this infection and the absence of direct cytopathic effect from infection in most cases. However, these findings may also suggest that the latest time point of this study (day 12) was too early to see significant lesions and that the course of this infection may be protracted.

One previous study/report in macaques infected with two strains of JUNV suggests some of the most severe lesions (i.e. polioencephalomyelitis) may occur at a much later time point, day 20 or later, and there is lesion variation with strain of virus [17]. It would be informative to study infection at day 15 and 20, or perhaps even later, if supported by survival, in specific organs such as the lymphoid organs, lungs, heart, brain, eye, adrenal glands and perhaps the liver. The lesions in the nervous system and adrenal gland would be particularly interesting to investigate at later time points given that lesions have been reported in the adrenal gland of guinea pigs in previous GTOV studies at the day 12–14 time point (adrenal necrosis with virus localization) [17] and in the brain of macaques infected with JUNV at much later time points (i.e. days 40 and 55) [17]. Further investigation of infection in autonomic ganglia (i.e. nervous plexi in the intestinal tract and ganglia adjacent to the adrenal gland) would also be instructive as similar findings have been previously reported to those observed in this study. This includes autonomic ganglioneuritis in rhesus macaques infected with JUNV, depending on strain (days 21–50) [17], and virus localized to ganglia in GTOV infected guinea pigs [20].

Overall, the histologic lesions in many organs, aside from lymphoid organs, were mild and nonspecific and many of the findings support those previously reported in other studies. This is especially true in the in lymphoid organs and bone marrow but also in other organs such as the adrenal gland [20,21,41,42]. However, there are also important distinctions such as the occurrence of CNS disease and lung lesions [41,43]. Much of this variation may be associated with differences in study design such as viral strain (i.e. *Junin*—Romero versus XJ or other), guinea pig strain (Hartley versus strain 13), route of administration (IP versus aerosol or SQ) and day of sampling (i.e. after day 12, the latest time point of this study). Viral labeling in multiple organs, including in the endothelium and various other regions such as the pulmonary and gastrointestinal epithelium has also been previously reported in either GTOV and/or JUNV infection models, along with viral titers in many organs [20,42]. In human cases of NW arenavirus infection, lymphoid tissue as well as the lung develop the highest virus titers. In addition to high levels of viral antigen in cells of the spleen and lymph node, viral antigen is also present in circulating mononuclear cells [16,44]. Virus has also been isolated from the adrenal glands, kidneys, liver and brain of patients at autopsy [13]. This study serves to further reinforce those previous findings, substantiates the guinea pig as an optimal model for studying NW arenavirus infection and importantly, provides a detailed comparison of two different NW arenaviruses, documenting the striking similarities in pathology and viral RNA distribution between the two viruses. These findings also support the previous clinical observations reported following infection with the two viruses [9] and coincides with their presence in the same phylogenetic lineage of the Tacaribe complex despite being geographically distinct and having different reservoirs/animal hosts [5,45].

The most pronounced differences between observations in JUNV versus GTOV infected guinea pigs were often observed in day 10 and 12 animals for ISH/viral RNA labeling in various organs. These differences can be visualized in ISH scoring graphs (Figs 4 and 6). While this may indicate a difference in infection properties between viruses it could also reflect differences in probe specificity in the ISH procedure between viruses and may not necessarily be reflective of infection dynamics. Organs in which there was higher intensity of viral RNA labeling in days 10 and/or 12 in the JUNV group included the spleen, bone marrow, eye, lung, liver, gallbladder, stomach and small intestine. Differences in histologic lesion severity or frequency between groups were often subtle and infrequent. These differences included more severe lymphocyte apoptosis/necrosis in lymph nodes of the GTOV infected animals but more severe lymphocyte apoptosis/necrosis in the spleen of JUNV infected animals. More severe necrosis/apoptosis in the bone marrow was observed in JUNV infected day 12 animals and lesions in the choroid plexus were also more common in this group and time pojnt. Additional differences include more consistent alveolar lung inflammation in the JUNV infected animals, and hemorrhage in the cortex of the adrenal gland only identified in day 12 JUNV-infected animals but not GTOV-infected animals. In general, a very similar constellation of histologic lesions and pattern of viral RNA labeling was present in both groups. The differences in histologic lesions and RNA labeling were mild and, in some cases subtle. Even so, these observations may suggest there are differences in pathogenicity and virulence which may help explain some of the differences in clinical signs between the two viruses that have been described [9]. Nonetheless, absence of more striking differences in pathology and viral RNA distribution may also indicate that development of medical countermeasures for one virus may be effective against others in this lineage.

Arenaviruses, as with many other causes of viral hemorrhagic fever, lack effective and approved vaccines and therapeutics. A protective vaccine is available for JUNV but is not FDA approved and has limited distribution [3,46]. Immune plasma therapy is effective against AHF, but it must be administered during the prodromal phase, within eight days of initial symptom onset [3,7]. Protective vaccines are not available for the other arenaviruses and JUNV antibodies do not offer cross protection against other NW arenaviruses [3,47], although infection with attenuated strains of NW arenavirus (i.e. MACV) have been shown to provide cross protection against other strains (i.e. GTOV) [48]. Nonetheless, even with effective antibody/immune plasma therapy for AHF, a small subset of treated patients develop long term neurological sequelae [5,7], emphasizing the need for additional and more widely available therapeutics. Effective therapeutics for many of the other pathogenic NW arenaviruses are unavailable, although work is ongoing in this area [7,46–48].

Antiviral therapies such as Faviparavir and Ribavarin have shown promise against arenavirus infection [3,7,49,50]. Additionally, some therapies exhibit limited cross protection between NW arenaviruses [48,51,52], but much of the work focused on Lassa virus and additional studies are necessary to determine safety, efficacy and in some cases *in vivo* effectiveness against NW arenaviruses [53]. Other novel therapies such as antibody blockade of NW arenavirus receptor human transferrin receptor 1 (hTFR1), which is the receptor used by all the pathogenic clade B NW arenaviruses, has recently shown promise in preventing viral infection with the potential for cross protection [46]. Other noteworthy infection dynamics that may aid in therapeutic development have also been discovered and include other receptors (C-type lectins) for JUNV in addition to hTFR1. Studies have also shown that non-pathogenic clade C NW arenaviruses use a different receptor, α-Dystroglycan, which is the same receptor used by Lassa virus [2,5,6,54]. Others have illustrated and discussed how the ability of non-pathogenic NW arenaviruses, including those present in North America, to mutate the glycoprotein that interacts with hTFR1 may result in the occurrence of a new human pathogen [5,55], adding a sense of urgency to this research.

Moreover, cost effective models that recapitulate human disease are needed to facilitate a better understanding of disease pathogenesis in order to develop additional medical countermeasures with the potential to offer protection across the range of current and emerging pathogenic NW arenaviruses. Here we characterize and compare infection with two different NW arenaviruses in the guinea pig model and clearly demonstrate its ability to reproduce many aspects of human disease. The key features include immunosuppression through effects on lymphoid organs, infection of endothelium, and extensive viral RNA labeling, including in the CNS, with absence of histologic lesions in some organs.

## References

[1] Buchmeier MJdlT Juan-Carlos; Peters Clarence J. Arenaviridae: The Viruses and Their Replication. In: Knipe DMHPM, editor. Fields Virology. 2. 5th ed: Lippincott Williams & Wilkins; 2007. p. 1791–828.

[2] Shao J, Liang Y, Ly H. Human hemorrhagic Fever causing arenaviruses: molecular mechanisms contributing to virus virulence and disease pathogenesis. Pathogens. 2015;4(2):283–306. Epub 2015/05/27. doi: 10.3390/pathogens4020283 ; PubMed Central PMCID: PMC4493475. 26011826 PMC4493475

[3] Brisse ME, Ly H. Hemorrhagic Fever-Causing Arenaviruses: Lethal Pathogens and Potent Immune Suppressors. Front Immunol. 2019;10:372. Epub 2019/03/29. doi: 10.3389/fimmu.2019.00372 ; PubMed Central PMCID: PMC6424867. 30918506 PMC6424867

[4] Milazzo ML, Campbell GL, Fulhorst CF. Novel arenavirus infection in humans, United States. Emerg Infect Dis. 2011;17(8):1417–20. Epub 2011/08/02. doi: 10.3201/eid1708.110285 ; PubMed Central PMCID: PMC3381580. 21801618 PMC3381580

[5] Sarute N, Ross SR. New World Arenavirus Biology. Annu Rev Virol. 2017;4(1):141–58. Epub 2017/06/25. doi: 10.1146/annurev-virology-101416-042001 ; PubMed Central PMCID: PMC7478856. 28645238 PMC7478856

[6] Hallam SJ, Koma T, Maruyama J, Paessler S. Review of Mammarenavirus Biology and Replication. Front Microbiol. 2018;9:1751. Epub 2018/08/21. doi: 10.3389/fmicb.2018.01751 ; PubMed Central PMCID: PMC6085440. 30123198 PMC6085440

[7] Enria DA, Briggiler AM, Sanchez Z. Treatment of Argentine hemorrhagic fever. Antiviral Res. 2008;78(1):132–9. Epub 2007/12/07. doi: 10.1016/j.antiviral.2007.10.010 ; PubMed Central PMCID: PMC7144853. 18054395 PMC7144853

[8] (NCEZID) NCfEaZID. Bioterrorism Agents/Diseases: Centers for Disease Control and Prevention; 2018 [updated 4 April 2018; cited 2022 1 September 2022]. Available from: https://emergency.cdc.gov/agent/agentlist-category.asp#catdef.

[9] Charrel RN, de Lamballerie X. Arenaviruses other than Lassa virus. Antiviral Res. 2003;57(1–2):89–100. Epub 2003/03/05. doi: 10.1016/s0166-3542(02)00202-4 . 12615305

[10] Peters CJ. Human infection with arenaviruses in the Americas. Curr Top Microbiol Immunol. 2002;262:65–74. Epub 2002/05/04. doi: 10.1007/978-3-642-56029-3_3 . 11987808

[11] Moraz ML, Kunz S. Pathogenesis of arenavirus hemorrhagic fevers. Expert Rev Anti Infect Ther. 2011;9(1):49–59. Epub 2010/12/22. doi: 10.1586/eri.10.142 . 21171877

[12] de Manzione N, Salas RA, Paredes H, Godoy O, Rojas L, Araoz F, et al. Venezuelan hemorrhagic fever: clinical and epidemiological studies of 165 cases. Clin Infect Dis. 1998;26(2):308–13. Epub 1998/03/21. doi: 10.1086/516299 . 9502447

[13] Elsner B, Schwarz E, Mando OG, Maiztegui J, Vilches A. Pathology of 12 fatal cases of Argentine hemorrhagic fever. Am J Trop Med Hyg. 1973;22(2):229–36. Epub 1973/03/01. . 4688419

[14] Schattner M, Rivadeneyra L, Pozner RG, Gomez RM. Pathogenic mechanisms involved in the hematological alterations of arenavirus-induced hemorrhagic fevers. Viruses. 2013;5(1):340–51. Epub 2013/01/23. doi: 10.3390/v5010340 ; PubMed Central PMCID: PMC3564124. 23337384 PMC3564124

[15] Salas R, de Manzione N, Tesh RB, Rico-Hesse R, Shope RE, Betancourt A, et al. Venezuelan haemorrhagic fever. Lancet. 1991;338(8774):1033–6. Epub 1991/10/26. doi: 10.1016/0140-6736(91)91899-6 . 1681354

[16] Gonzalez PH, Cossio PM, Arana R, Maiztegui JI, Laguens RP. Lymphatic tissue in Argentine hemorrhagic fever. Pathologic features. Arch Pathol Lab Med. 1980;104(5):250–4. Epub 1980/05/01. . 6154445

[17] Green DE, Mahlandt BG, McKee KT Jr., Experimental Argentine hemorrhagic fever in rhesus macaques: virus-specific variations in pathology. J Med Virol. 1987;22(2):113–33. Epub 1987/06/01. doi: 10.1002/jmv.1890220203 . 3039051

[18] Kolokoltsova OA, Yun NE, Poussard AL, Smith JK, Smith JN, Salazar M, et al. Mice lacking alpha/beta and gamma interferon receptors are susceptible to junin virus infection. J Virol. 2010;84(24):13063–7. Epub 2010/10/12. doi: 10.1128/JVI.01389-10 ; PubMed Central PMCID: PMC3004311. 20926559 PMC3004311

[19] McKee KT, Jr., Oro JG, Kuehne AI, Spisso JA, Mahlandt BG. Candid No. 1 Argentine hemorrhagic fever vaccine protects against lethal Junin virus challenge in rhesus macaques. Intervirology. 1992;34(3):154–63. Epub 1992/01/11. doi: 10.1159/000150276 . 1338783

[20] Hall WC, Geisbert TW, Huggins JW, Jahrling PB. Experimental infection of guinea pigs with Venezuelan hemorrhagic fever virus (Guanarito): a model of human disease. Am J Trop Med Hyg. 1996;55(1):81–8. Epub 1996/07/01. doi: 10.4269/ajtmh.1996.55.81 . 8702027

[21] Yun NE, Linde NS, Dziuba N, Zacks MA, Smith JN, Smith JK, et al. Pathogenesis of XJ and Romero strains of Junin virus in two strains of guinea pigs. Am J Trop Med Hyg. 2008;79(2):275–82. Epub 2008/08/12. ; PubMed Central PMCID: PMC2700623. 18689636 PMC2700623

[22] Bell TM, Bunton TE, Shaia CI, Raymond JW, Honnold SP, Donnelly GC, et al. Pathogenesis of Bolivian Hemorrhagic Fever in Guinea Pigs. Vet Pathol. 2016;53(1):190–9. Epub 2015/07/04. doi: 10.1177/0300985815588609 . 26139838

[23] Kenyon RH, Green DE, Maiztegui JI, Peters CJ. Viral strain dependent differences in experimental Argentine hemorrhagic fever (Junin virus) infection of guinea pigs. Intervirology. 1988;29(3):133–43. Epub 1988/01/01. doi: 10.1159/000150039 . 2846464

[24] Golden JW, Maes P, Kwilas SA, Ballantyne J, Hooper JW. Glycoprotein-Specific Antibodies Produced by DNA Vaccination Protect Guinea Pigs from Lethal Argentine and Venezuelan Hemorrhagic Fever. J Virol. 2016;90(7):3515–29. Epub 2016/01/23. doi: 10.1128/JVI.02969-15 ; PubMed Central PMCID: PMC4794662. 26792737 PMC4794662

[25] Webb PA, Johnson KM, Mackenzie RB. The measurement of specific antibodies in Bolivian hemorrhagic fever by neutralization of virus plaques. Proc Soc Exp Biol Med. 1969;130(3):1013–9. Epub 1969/03/01. doi: 10.3181/00379727-130-33711 . 4975291

[26] Berkebile ZW, Putri DS, Abrahante JE, Seelig DM, Schleiss MR, Bierle CJ. The Placental Response to Guinea Pig Cytomegalovirus Depends Upon the Timing of Maternal Infection. Front Immunol. 2021;12:686415. Epub 2021/07/03. doi: 10.3389/fimmu.2021.686415 ; PubMed Central PMCID: PMC8239309. 34211475 PMC8239309

[27] Willard-Mack CL, Elmore SA, Hall WC, Harleman J, Kuper CF, Losco P, et al. Nonproliferative and Proliferative Lesions of the Rat and Mouse Hematolymphoid System. Toxicol Pathol. 2019;47(6):665–783. Epub 2019/09/19. doi: 10.1177/0192623319867053 ; PubMed Central PMCID: PMC6752743. 31526133 PMC6752743

[28] DH BSGSP. Pathology of Laboratory Rodents and Rabbits. 4th ed. Ames IA: Wiley Blackwell; 2016 15 January 2016. 384 p.

[29] Cuba-Caparo A, Myers DM, Germino NI. Focal hepatic necrosis in clinically normal guinea pigs: bacteriological and pathological studies. J Comp Pathol. 1977;87(3):441–50. Epub 1977/07/01. doi: 10.1016/0021-9975(77)90033-0 908770

[30] Maeda H KH, Sato K, Satake S, Izumi H, Miyajima H. Spontaneous Multiple Focal to Massive Hepatic Necrosis in Guinea Pigs. J Toxicol Pathol. 2000;13:207~12

[31] Bell TM, Shaia CI, Bearss JJ, Mattix ME, Koistinen KA, Honnold SP, et al. Temporal Progression of Lesions in Guinea Pigs Infected With Lassa Virus. Vet Pathol. 2017;54(3):549–62. Epub 2017/04/26. doi: 10.1177/0300985816677153 . 28438110

[32] Fletcher AL, Acton SE, Knoblich K. Lymph node fibroblastic reticular cells in health and disease. Nat Rev Immunol. 2015;15(6):350–61. Epub 2015/05/23. doi: 10.1038/nri3846 ; PubMed Central PMCID: PMC5152733. 25998961 PMC5152733

[33] Groseth A, Hoenen T, Weber M, Wolff S, Herwig A, Kaufmann A, et al. Tacaribe virus but not junin virus infection induces cytokine release from primary human monocytes and macrophages. PLoS Negl Trop Dis. 2011;5(5):e1137. Epub 2011/05/17. doi: 10.1371/journal.pntd.0001137 ; PubMed Central PMCID: PMC3091837. 21572983 PMC3091837

[34] Meyer B, Ly H. Inhibition of Innate Immune Responses Is Key to Pathogenesis by Arenaviruses. J Virol. 2016;90(8):3810–8. Epub 2016/02/13. doi: 10.1128/JVI.03049-15 ; PubMed Central PMCID: PMC4810556. 26865707 PMC4810556

[35] Levis SC, Saavedra MC, Ceccoli C, Feuillade MR, Enria DA, Maiztegui JI, et al. Correlation between endogenous interferon and the clinical evolution of patients with Argentine hemorrhagic fever. J Interferon Res. 1985;5(3):383–9. Epub 1985/01/01. doi: 10.1089/jir.1985.5.383 . 4056485

[36] Versteeg GA, Garcia-Sastre A. Viral tricks to grid-lock the type I interferon system. Curr Opin Microbiol. 2010;13(4):508–16. Epub 2010/06/12. doi: 10.1016/j.mib.2010.05.009 ; PubMed Central PMCID: PMC2920345. 20538505 PMC2920345

[37] Weber F, Haller O. Viral suppression of the interferon system. Biochimie. 2007;89(6–7):836–42. Epub 2007/03/06. doi: 10.1016/j.biochi.2007.01.005 ; PubMed Central PMCID: PMC7126635. 17336442 PMC7126635

[38] Marta RF, Montero VS, Hack CE, Sturk A, Maiztegui JI, Molinas FC. Proinflammatory cytokines and elastase-alpha-1-antitrypsin in Argentine hemorrhagic fever. Am J Trop Med Hyg. 1999;60(1):85–9. Epub 1999/02/13. doi: 10.4269/ajtmh.1999.60.85 . 9988328

[39] Kunz S. The role of the vascular endothelium in arenavirus haemorrhagic fevers. Thromb Haemost. 2009;102(6):1024–9. Epub 2009/12/08. doi: 10.1160/TH09-06-0357 . 19967131

[40] Gomez RM, Pozner RG, Lazzari MA, D’Atri LP, Negrotto S, Chudzinski-Tavassi AM, et al. Endothelial cell function alteration after Junin virus infection. Thromb Haemost. 2003;90(2):326–33. Epub 2003/07/31. doi: 10.1160/TH02-09-0043 . 12888881

[41] Kenyon RH, Green DE, Eddy GA, Peters CJ. Treatment of junin virus-infected guinea pigs with immune serum: development of late neurological disease. J Med Virol. 1986;20(3):207–18. Epub 1986/11/01. doi: 10.1002/jmv.1890200303 . 3023540

[42] Vela E. Animal models, prophylaxis, and therapeutics for arenavirus infections. Viruses. 2012;4(9):1802–29. Epub 2012/11/22. doi: 10.3390/v4091802 ; PubMed Central PMCID: PMC3499831. 23170184 PMC3499831

[43] Laguens RM, Avila MM, Samoilovich SR, Weissenbacher MC, Laguens RP. Pathogenicity of an attenuated strain (XJCl3) of Junin virus. Morphological and virological studies in experimentally infected guinea pigs. Intervirology. 1983;20(4):195–201. Epub 1983/01/01. doi: 10.1159/000149392 . 6317604

[44] Ambrosio AM, Enria DA, Maiztegui JI. Junin virus isolation from lympho-mononuclear cells of patients with Argentine hemorrhagic fever. Intervirology. 1986;25(2):97–102. Epub 1986/01/01. doi: 10.1159/000149662 . 3013799

[45] Bowen MD, Peters CJ, Nichol ST. Phylogenetic analysis of the Arenaviridae: patterns of virus evolution and evidence for cospeciation between arenaviruses and their rodent hosts. Mol Phylogenet Evol. 1997;8(3):301–16. Epub 1998/01/07. doi: 10.1006/mpev.1997.0436 . 9417890

[46] Ferrero S, Flores MD, Short C, Vazquez CA, Clark LE, Ziegenbein J, et al. Antibody-Based Inhibition of Pathogenic New World Hemorrhagic Fever Mammarenaviruses by Steric Occlusion of the Human Transferrin Receptor 1 Apical Domain. J Virol. 2021;95(17):e0186820. Epub 2021/06/17. doi: 10.1128/JVI.01868-20 ; PubMed Central PMCID: PMC8354235. 34132574 PMC8354235

[47] Leske A, Wassmann I, Schnepel K, Shifflett K, Holzerland J, Bostedt L, et al. Assessing cross-reactivity of Junin virus-directed neutralizing antibodies. Antiviral Res. 2019;163:106–16. Epub 2019/01/23. doi: 10.1016/j.antiviral.2019.01.006 . 30668977

[48] Golden JW, Beitzel B, Ladner JT, Mucker EM, Kwilas SA, Palacios G, et al. An attenuated Machupo virus with a disrupted L-segment intergenic region protects guinea pigs against lethal Guanarito virus infection. Sci Rep. 2017;7(1):4679. Epub 2017/07/07. doi: 10.1038/s41598-017-04889-x ; PubMed Central PMCID: PMC5498534. 28680057 PMC5498534

[49] Charrel RN, Coutard B, Baronti C, Canard B, Nougairede A, Frangeul A, et al. Arenaviruses and hantaviruses: from epidemiology and genomics to antivirals. Antiviral Res. 2011;90(2):102–14. Epub 2011/03/02. doi: 10.1016/j.antiviral.2011.02.009 . 21356244

[50] Gowen BB, Westover JB, Sefing EJ, Van Wettere AJ, Bailey KW, Wandersee L, et al. Enhanced protection against experimental Junin virus infection through the use of a modified favipiravir loading dose strategy. Antiviral Res. 2017;145:131–5. Epub 2017/08/07. doi: 10.1016/j.antiviral.2017.07.019 ; PubMed Central PMCID: PMC5580092. 28780425 PMC5580092

[51] Lopez N, Scolaro L, Rossi C, Jacamo R, Candurra N, Pujol C, et al. Homologous and heterologous glycoproteins induce protection against Junin virus challenge in guinea pigs. J Gen Virol. 2000;81(Pt 5):1273–81. Epub 2000/04/18. doi: 10.1099/0022-1317-81-5-1273 . 10769070

[52] Sanchez A, Pifat DY, Kenyon RH, Peters CJ, McCormick JB, Kiley MP. Junin virus monoclonal antibodies: characterization and cross-reactivity with other arenaviruses. J Gen Virol. 1989;70 (Pt 5):1125–32. Epub 1989/05/01. doi: 10.1099/0022-1317-70-5-1125 . 2471803

[53] Delang L, Abdelnabi R, Neyts J. Favipiravir as a potential countermeasure against neglected and emerging RNA viruses. Antiviral Res. 2018;153:85–94. Epub 2018/03/11. doi: 10.1016/j.antiviral.2018.03.003 . 29524445

[54] Martinez MG, Bialecki MA, Belouzard S, Cordo SM, Candurra NA, Whittaker GR. Utilization of human DC-SIGN and L-SIGN for entry and infection of host cells by the New World arenavirus, Junin virus. Biochem Biophys Res Commun. 2013;441(3):612–7. Epub 2013/11/05. doi: 10.1016/j.bbrc.2013.10.106 ; PubMed Central PMCID: PMC4096786. 24183720 PMC4096786

[55] Zong M, Fofana I, Choe H. Human and host species transferrin receptor 1 use by North American arenaviruses. J Virol. 2014;88(16):9418–28. Epub 2014/06/13. doi: 10.1128/JVI.01112-14 ; PubMed Central PMCID: PMC4136298. 24920811 PMC4136298

